# The Hyphal-Associated Adhesin and Invasin Als3 of *Candida albicans* Mediates Iron Acquisition from Host Ferritin

**DOI:** 10.1371/journal.ppat.1000217

**Published:** 2008-11-21

**Authors:** Ricardo S. Almeida, Sascha Brunke, Antje Albrecht, Sascha Thewes, Michael Laue, John E. Edwards, Scott G. Filler, Bernhard Hube

**Affiliations:** 1 Department of Microbial Pathogenicity Mechanisms, Leibniz Institute for Natural Product Research and Infection Biology – Hans Knoell Institute, Jena, Germany; 2 Friedrich Schiller University Jena, Jena, Germany; 3 Department of Biology, Chemistry and Pharmacy, Institute for Biology – Microbiology, Free University Berlin, Berlin, Germany; 4 Centre for Biological Safety 4 (ZBS4), Robert Koch Institute, Berlin, Germany; 5 David Geffen School of Medicine, University of California Los Angeles, Los Angeles, California, United States of America; 6 Department of Medicine, Los Angeles Biomedical Research Institute, Harbor-UCLA Medical Center, Torrance, California, United States of America; Carnegie Mellon University, United States of America

## Abstract

Iron sequestration by host iron-binding proteins is an important mechanism of resistance to microbial infections. Inside oral epithelial cells, iron is stored within ferritin, and is therefore not usually accessible to pathogenic microbes. We observed that the ferritin concentration within oral epithelial cells was directly related to their susceptibility to damage by the human pathogenic fungus, *Candida albicans*. Thus, we hypothesized that host ferritin is used as an iron source by this organism. We found that *C. albicans* was able to grow on agar at physiological pH with ferritin as the sole source of iron, while the baker's yeast *Saccharomyces cerevisiae* could not. A screen of *C. albicans* mutants lacking components of each of the three known iron acquisition systems revealed that only the reductive pathway is involved in iron utilization from ferritin by this fungus. Additionally, *C. albicans* hyphae, but not yeast cells, bound ferritin, and this binding was crucial for iron acquisition from ferritin. Transcriptional profiling of wild-type and hyphal-defective *C. albicans* strains suggested that the *C. albicans* invasin-like protein Als3 is required for ferritin binding. Hyphae of an *Δals3* null mutant had a strongly reduced ability to bind ferritin and these mutant cells grew poorly on agar plates with ferritin as the sole source of iron. Heterologous expression of Als3, but not Als1 or Als5, two closely related members of the Als protein family, allowed *S. cerevisiae* to bind ferritin. Immunocytochemical localization of ferritin in epithelial cells infected with *C. albicans* showed ferritin surrounding invading hyphae of the wild-type, but not the *Δals3* mutant strain. This mutant was also unable to damage epithelial cells *in vitro*. Therefore, *C. albicans* can exploit iron from ferritin via morphology dependent binding through Als3, suggesting that this single protein has multiple virulence attributes.

## Author Summary

Iron is an essential nutrient for all microbes. Many human pathogenic microbes have developed sophisticated strategies to acquire iron from the host as most compartments in the body contain little free iron. For example, in oral epithelial cells intracellular iron is bound to ferritin, a protein that is highly resistant to microbial attack. In fact, no microorganism has so far been shown to directly exploit ferritin as an iron source during interaction with host cells. This study demonstrates that the pathogenic fungus *Candida albicans* can use ferritin as the sole source of iron. Most intriguingly, *C. albicans* binds ferritin via a receptor that is only exposed on invasive hyphae. This receptor is Als3, which is a member of the Als-protein family. Als3 was previously demonstrated to be an adhesin with invasin-like properties. Mutants lacking Als3 failed to bind ferritin, grew poorly with ferritin as an iron source and were unable to damage epithelial cells. Strains of the baker's yeast expressing *C. albicans* Als3, but not two closely related proteins, Als1 or Als5, were able to bind ferritin. Therefore, *C. albicans* uses an additional morphology specific and unique iron uptake strategy based on ferritin while invading into host cells where ferritin is located.

## Introduction

Iron is an essential element for virtually all organisms, ranging from microbes to multicellular animals. Higher organisms can sequester iron using high-affinity iron-binding molecules, so that it is unavailable to microorganisms. Iron sequestration provides a natural resistance to infections which has been described as “nutritional immunity” [1].

Successful microbial pathogens have developed multiple iron acquisition and uptake systems (reviewed in [2],[3]). These systems include enzymes for reduction and oxidization of iron ions (Fe^2+^ or Fe^3+^), high-affinity permeases for iron transport, chelators (siderophores) and uptake systems for siderophores. In the human body, the majority of iron is bound to iron-containing proteins with physiological functions (for example heme proteins such as hemoglobin), iron-binding transport proteins (transferrin), antimicrobial proteins (lactoferrin), or cellular iron storage proteins (ferritin). With the exception of ferritin, each of these proteins has been reported to serve as an iron source for some pathogenic microbes. These iron sources are exploited via direct binding, degradation, and/or uptake [4]–[13].

In mammalian cells, extracellular ferric iron is bound by apotransferrin (transferrin without iron). The binding of apotransferrin to two ferric iron molecules (holotransferrin, hTF) increases by two-fold its affinity for the transferrin receptor (TFR) present on the surface of virtually all mammalian cells. Following endocytosis of the hTF-TFR complex into the early endosome, acidification to low pH (pH 5.6) results in the release of iron from holotransferrin. The released ferrous iron is then transported to the cytoplasm by the divalent metal transporter (DMT1) and either used for cellular metabolism or stored within ferritin. The resulting apotransferrin is recycled to the cell surface and released at physiological pH (7.4) [14]–[16].

Ferritin is the main intracellular storage protein for iron (reviewed in [17]), containing approximately 30% of the total human body iron (66% is bound to hemoglobin). Ferritin consist of a 24-subunit protein shell of approximately 500 kDa. One ferritin molecule can contain up to 4,500 Fe^3+^ ions. The quaternary structure of ferritin is dissociated at acidic pH [18]. Its intracellular concentration can be increased by addition of exogenous iron and decreased by addition of an iron chelator [19]. Under iron-limiting conditions, cytosolic ferritin is autophagocytosed and subsequently degraded within acidic lysosomes [19],[20] and the iron becomes available to the cell. Outside of lysosomes, ferritin is an extremely robust and stable protein which seems to be resistant to all known microbial activities. In fact, the only microorganism that has so far been shown experimentally to exploit holoferritin as an iron source during interaction with host cells is *Neisseria meningitidis* [21]. *N. meningitidis* bacteria can trigger degradation of cytosolic ferritin within infected epithelial cells by manipulating the cellular machinery and lysosomal activity [22].

To secure sufficient iron availability whilst avoiding toxicity by iron mediated processes, a tight regulation of iron metabolism is essential. Some pathogenic microbes seem to have linked the availability of iron with expression of virulence attributes. For example, the expression of virulence genes in *Listeria monocytogenes* was found to be positively controlled by iron limitation [23] and infections with *Mycobacterium tuberculosis* were reported to be more fatal when iron was accessible [24]. In pathogenic *Escherichia coli* strains, more than 90 genes involved in iron acquisition and several other cellular functions such as chemotaxis, respiration, DNA synthesis, glycolysis and the tricarboxylic acid cycle are co-regulated by the global iron-dependent regulator *FUR* (**F**erric-**U**ptake **R**egulator protein) [2]. The fungus, *Cryptococcus neoformans* has recently been shown to co-regulate iron uptake mechanisms with two key virulence properties, capsule formation and melanin production [25],[26]. Such coordinated regulation indicates that sensing the low iron content of the host environment is a key signal for pathogenic microbes to initiate adaptation to the host and express factors such as toxins and siderophores to facilitate access to host iron sources [27],[28].

*Candida albicans* is a polymorphic yeast which is part of the normal microbial flora of humans. The fungus lives as a harmless commensal on mucosal surfaces in healthy individuals, but can cause several types of infections in predisposed patients, ranging from superficial to life threatening disease [29]. During infection, *C. albicans* can grow in almost all body sites and organs, indicating an astonishing metabolic flexibility, a high level of stress resistance and effective immune evasion strategies. One of the key features of *C. albicans* is its ability to grow in different morphological forms – either as ovoid yeast, a filamentous hyphal form or as pseudohyphae [30]. Although the yeast form appears to be important for dissemination [31], the hyphal form is of crucial importance for cell and tissue invasion [32]–[34]. Furthermore, genes known or proposed to be associated with adhesion, invasion, extracellular hydrolytic activity, detoxification or as yet unknown functions (*HWP1*, *ALS3*, *SAP4-6*, *SOD5*, *HYR1*, *ECE1*) are co-regulated with the yeast-to-hyphal transition [35]–[40]. Both cellular morphology and expression of hyphal associated genes are tightly regulated by a network of signal transduction pathways (including MAP kinase, cAMP and Rim101 pathways [30],[41]) and transcriptional activators and repressors such as Efg1, Tec1, Bcr1, Tup1 and Nrg1 [42]–[46].

*C. albicans* adaptation to the host environment is also reliant on a large number of genes associated with iron acquisition [47]. These genes contribute to the three known iron acquisition systems of *C. albicans*: (1) Uptake and utilization of iron from hemoglobin is mediated by Rbt5 and Hmx1 [13],[48],[49]. *In vitro* data have shown that Rbt5 is a hemoglobin receptor that binds hemoglobin on the surface. This binding seems to induce expression of *HMX1*, which encodes a heme oxygenase. This activity is essential for iron utilization from heme [49]. (2) Iron in siderophores is taken up via the siderophore transporter, Sit1 [50],[51]. *C. albicans* siderophore production had been demonstrated by biochemical assays in earlier studies [52],[53]. However, in contrast to *Aspergillus fumigatus* [54], genes encoding factors of a possible siderophore production pathway have not been discovered in the *C. albicans* genome [47]. Nevertheless, Sit1 can mediate uptake of a range of heterologous siderophores from other organisms and other iron complexes [50], [55]–[57]. (3) To use free iron from the environment, iron from transferrin, and possibly iron from other so far unknown sources, *C. albicans* uses the reductive uptake system. This system is located in the plasma membrane and has three components. The first component consists of ferric reductases. At least two surface ferric reductases, which are able to reduce insoluble extracellular ferric (Fe^3+^) ions into soluble Fe^2+^ ions, have been described [7],[58],[59]. In addition thirteen homologous genes, putatively encoding other ferric reductases have been identified in the *C. albicans* genome (http://www.Candidagenome.org). The second component consists of multicopper oxidase. Reduced ferrous iron generated by surface reductase activity can be toxic due to spontaneous generation of free radicals. However, Fe^2+^ can also be oxidized to Fe^3+^ by multicopper oxidase activity and thus preventing the production of toxic free radicals [60],[61]. The *C. albicans* genome contains five putative multicopper oxidase genes [62]. Due to the copper requirement of the oxidase activity, the intracellular copper transporter Ccc2 is essential for this reductive pathway [63]. The third component consists of iron permeases. These form a protein complex with multicopper oxidases and transport Fe^3+^ into the cell. *C. albicans* has two iron permeases that are encoded by two highly homologous genes. The high-affinity iron permease gene, *FTR1* is induced by iron deprivation and the low-affinity iron permease gene, *FTR2* is induced when higher levels of iron are available [64].

All three iron acquisition systems appear to be independent from each other and so far only Ftr1 has been shown to be crucial for *C. albicans* virulence in an experimental animal model of infection [64]. However, it is unclear which iron sources are used during the different types of *C. albicans* infection and within different anatomical sites. Recent *in vitro* and *in vivo* transcriptional profiling experiments have shown that *C. albicans* gene expression is tissue specific [33],[34],[65]. Since the relative proportion of iron-containing proteins differs among the different anatomical sites, we propose that usage of iron by *C. albicans* is niche specific.

Within the oral cavity, extracellular iron is bound mostly to lactoferrin in saliva and intracellular iron is stored in ferritin. However, oral infections by *C. albicans* are frequent, suggesting that *C. albicans* must be able to exploit the host iron resources of the oral cavity. We observed that genes encoding the high-affinity reductive iron uptake system of *C. albicans* are up-regulated during oral infections in patients [33]. Also, *C. albicans* causes greater damage to oral epithelial cells that contain a high concentration of ferritin (this study). Therefore, we hypothesized that host ferritin may be used as an iron source by this organism. Here we show that *C. albicans* can utilize iron from ferritin via morphology dependent binding through the adhesin and invasin Als3, suggesting that this single protein has multiple virulence attributes.

## Results

### The Ferritin Content of Epithelial Cells Influences the Extent of Cellular Damage Caused by *C. albicans*

**Figure 1:**
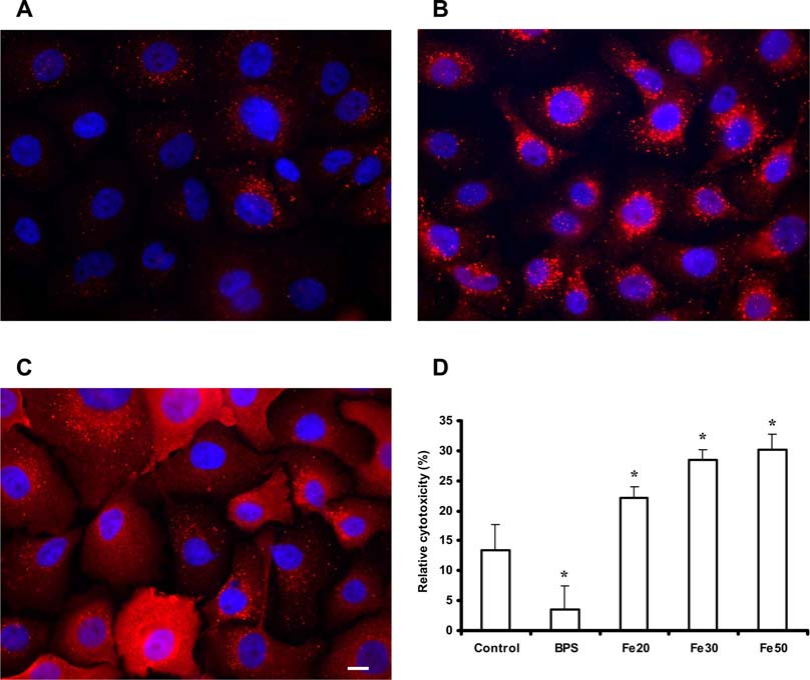
The ferritin content of host epithelial cells influences the cell damage by *C. albicans*. The ferritin content was monitored using immunofluorescence (red, antibody staining for ferritin; blue, nuclei staining with DAPI). Following the treatments described in (A), (B) and (C), the monolayers were washed and incubated for 8 h in serum-free RPMI with 10^6^ iron starved *C. albicans* (SC5314) cells. Cell damage was quantified by monitoring the release of epithelial LDH into the medium. (A) Monolayer incubated for 24 h in serum-free RPMI with 50 µM BPS. (B) Monolayer incubated 24 h in RPMI with 10% FBS (control). (C) Monolayer incubated for 24 h in RPMI with 10% FBS and 50 µM iron chloride. Bar indicates 10 µm. (D) Cell damage caused by *C. albicans*, calculated in relative cytotoxicity (%). Control, monolayers preincubated in normal cell culture medium (RPMI 1640 medium with 10% FBS); BPS, monolayers preincubated in serum-free RPMI with 50 µM BPS (iron chelator); Fe20, Fe30 and Fe50, monolayers preincubated in cell culture medium with 20, 30 and 50 µM ferric iron respectively. The experiment was performed twice in duplicates. *, significant difference compared to the control (p<0.05).

*C. albicans* can attach to and proliferate on oral epithelial tissue and can invade and damage epithelial cells [66]. To elucidate which iron sources are exploited during growth on and invasion of oral epithelial cells and to determine how the availability of iron influences fungal-host cell interactions, we incubated oral epithelial cell monolayers in the presence of additional free iron or the iron chelator bathophenanthrolindisulphonic acid (BPS). This chelator sequesters extracellular, but not intracellular iron [67]. Through immunocytochemical localization of ferritin within epithelial cells, we found that addition of BPS caused a dramatic decrease in cellular ferritin within 24 hours of incubation (Figure 1A), in comparison to non-treated cells (Figure 1B). In contrast, addition of free iron to the medium increased the concentration of ferritin within the same time period (Figure 1C). The treatment with additional iron or the iron chelator itself did not cause cell damage, as monitored by measuring the release of epithelial lactate dehydrogenase (LDH) into the supernatant (not shown).

Next, ferritin enriched or depleted epithelial monolayers were incubated for 8 h with *C. albicans* in normal cell culture medium (serum-free RPMI1640) and cell damage caused by *C. albicans* was monitored by LDH release. The epithelial monolayers depleted of ferritin were significantly protected from damage in comparison to untreated monolayers (control) (Figure 1D). In contrast, ferritin enriched epithelial cells were significantly and dose dependently more susceptible to damage caused by *C. albicans* (Figure 1D). These observations suggested that the ferritin content of epithelial cells directly correlates with cell damage and opened up the possibility that *C. albicans* can use ferritin as an iron source.

### Depletion of Ferritin, but not Ferritin Saturation Influences Invasion of Epithelial Cells by *C. albicans*

**Figure 2:**
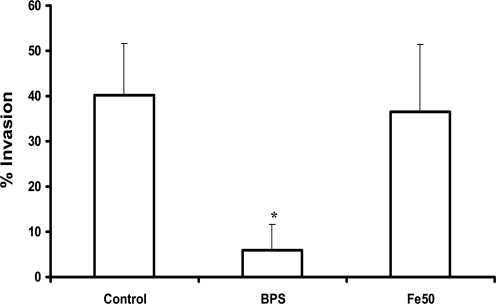
Invasion of ferritin depleted or enriched epithelial cells by *C. albicans*. Approximately 10^5^ iron starved *C. albicans* cells (SC5314) were co-incubated with ferritin depleted (BPS), ferritin enriched (Fe50) or non-treated (Control ) epithelial cells for 3 h. After fixation the samples were differentially stained and analyzed under the fluorescence microscope. The experiment was performed three times in duplicate. *, significant difference compared to non-treated epithelial cells (control) (p<0.001).

To clarify whether the observed increased or decreased cytotoxicity was due to either reduced or increased invasion of epithelial cells by *C. albicans*, we quantified invasion (after 3 h of co-incubation) in iron depleted versus iron saturated epithelial cells. Invasion of *C. albicans* into iron depleted epithelial cells was drastically reduced (Figure 2). It is known that *C. albicans* must invade oral epithelial cells to cause epithelial cell damage [68]. Therefore, the decreased epithelial cell invasion likely contributed to the reduced epithelial cell damage caused by iron depletion. In contrast, iron saturated epithelial cells were invaded at the same proportion as compared to untreated cells (Figure 2), even though *C. albicans* caused much more damage to these cells. These results suggest that the iron content of epithelial cell influences their susceptibility to damage by *C. albicans*, a mechanism that is at least partially independent of the extent of epithelial cell invasion.

### *C. albicans* Can Use Ferritin as the Sole Source of Iron *in vitro*

**Figure 3:**
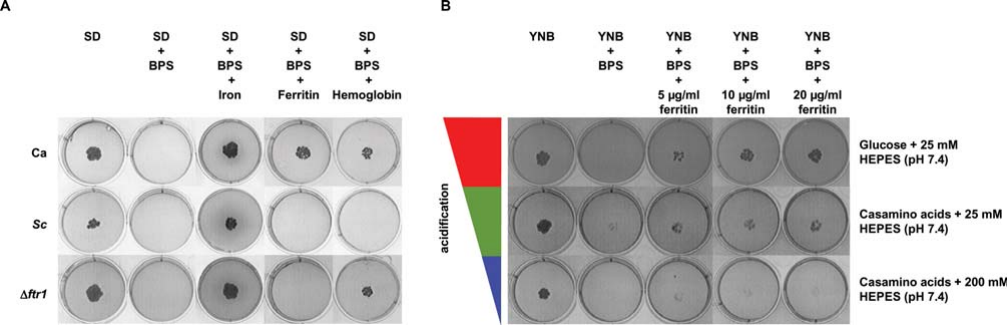
Usage of ferritin by *C. albicans* requires the reductive pathway and is mediated by acidification of the medium. (A) SD agar plates were adjusted to pH 7.4 with 25 mM HEPES buffer and incubated for 3 days at 37°C under 5% CO_2_ (Ca, *C. albicans* SC5314) or 30°C without CO_2_ (Sc, *S. cerevisiae* ATCC9763). Iron indicates 50 µM iron sulphate. Ferritin indicates 20 µg/ml ferritin. Hemoglobin indicates 20 µg/ml hemoglobin. (B) *C. albicans* wild-type (SC5314) cells were spotted on YNB agar with the addition of either glucose (SD) or casamino acids as a carbon source and buffered with 25 or 200 mM HEPES. BPS (iron chelator) was used to remove free iron from the media. The growth of *C. albicans* strains and *S. cerevisiae* on agar with different iron sources was repeated at least 3 times.

One explanation for the increased susceptibility of iron saturated epithelial cells to damage by *C. albicans* is that the organism uses epithelial cell ferritin as an iron source and is thereby able to produce more cytotoxic factors. To test whether *C. albicans* can use ferritin as an iron source *in vitro*, we incubated fungal cells on agar with ferritin as the sole iron source. By addition of BPS to the medium, we were able to remove any residual iron from the agar, medium or plastic surfaces. Only the addition of an external iron source allowed fungal growth under these conditions. Moreover, to minimize possible iron contamination of the ferritin solutions (not shown), we passed the ferritin through a column (Microcon YM-100, see Material and Methods) and washed it once with 5 mM HEPES buffer (pH 7.4) prior to use. Addition of free ferric iron, hemoglobin or ferritin to the agar promoted the growth of *C. albicans* at pH 7.4 (Figure 3A). In contrast, the baker's yeast *Saccharomyces cerevisiae*, known to be unable to grow with hemoglobin as the sole source of iron [13], only grew after addition of free iron to the medium (Figure 3A). However, *S. cerevisiae* was able to grow with ferritin when the initial pH of the medium was calibrated to pH 5.0 (not shown). This result suggested that the external pH of the medium influenced the bio-availability of iron from ferritin.

### The Use of Ferritin as the Sole Source of Iron *in vitro* Requires the Reductive Pathway and is Mediated by Acidification of the Medium

The ferritin protein shell is unstable at acidic pH [18]. Therefore, our finding that *S. cerevisiae* can utilize iron from ferritin at acidic, but not alkaline pH, suggested the possibility that *C. albicans* is able to release iron from this protein by active acidification of the medium. In fact, *C. albicans* was able to acidify a medium buffered with 25 mM HEPES (initial pH 7.4) during incubation with ferritin as sole source of iron as monitored by the pH indicator bromocresol green (Figure S1). To determine whether ferritin utilization is dependent on fungal-driven acidification, we substituted the glucose in the medium for casamino acids. This mixture of amino acids can be used as a carbon source by yeasts and avoids the acidification associated with glucose use [69],[70]. Furthermore, we stabilized the buffering capacity of the medium by the addition of HEPES buffer (pH 7.4) with increasing concentrations. As shown in Figure 3B, decreasing the capacity to acidify the medium, reduced the ability of *C. albicans* to grow with ferritin as a sole source of iron.

Next, we sought to determine which of the three known iron uptake systems of *C. albicans* are involved in iron acquisition from ferritin. Mutants lacking key genes of each iron acquisition system were screened for growth on ferritin agar plates. A mutant lacking the high-affinity permease Ftr1 was able to grow with free iron, hemoglobin, but not with ferritin as the sole iron source (Figure 3A; Table 1). Similarly, a mutant, lacking the copper transporter Ccc2, which is also essential for the reductive pathway, did not grow on ferritin plates (Figure S2; Table 1). In contrast to *S. cerevisiae*, *Δftr1* and *Δccc2* mutants did not grow on ferritin plates even when the initial pH was reduced to 5.0 (not shown). As expected, the *Δftr1+FTR1* and *Δccc2+CCC2* re-integrant strains grew similarly to the wild-type strain in the presence of ferritin (Figure S2). These observations suggest that the reductive pathway is essential for *C. albicans* to acquire iron from ferritin.

**Table 1:** Growth of different strains on ferritin agar plates.

	Wild-type	Reductive pathway		Siderophore and hemoglobin receptors		Aspartic proteases	
	CAF2-1	*Δftr1*	*Δccc2*	*Δsit1*	*Δrbt5*	*Δsap1-3*	*Δsap4-6*
SD	+	+	+	+	+	+	+
SD+BPS	−	−	−	−	−	−	−
SD+BPS+5 µg/ml ferritin	+	−	−	+	+	+	+

SD agar was buffered using 100 mM HEPES (pH 7.4). BPS, iron chelator. All plates were incubated for 3 days at 37°C under 5% CO_2_. See Figure S1 for details.

The *Δsit1* and *Δrbt5* mutants grew normally when ferritin was the sole iron source, indicating that *C. albicans* utilization of iron from ferritin is independent of the siderophore and hemoglobin uptake systems (Figure S2; Table 1).

We also investigated the possibility that aspartic proteases secreted by *C. albicans* could break down ferritin and release iron. The *Δsap1-3* and *Δsap4-6* triple-mutants grew similarly to wild-type cells on ferritin plates, suggesting that secreted aspartic proteases of *C. albicans* are not involved in liberating iron from ferritin (Figure S2; Table 1).

### Hyphal, but not Yeast Cells of *C. albicans* Can Bind Ferritin

Pathogenic microbes frequently utilize iron from host proteins by binding these molecules via specific receptors [4]–[8],[12],[13],[71]. Since our data showed that *C. albicans* can use ferritin as a sole source of iron, we investigated whether *C. albicans* can bind ferritin on its surface.

**Figure 4:**
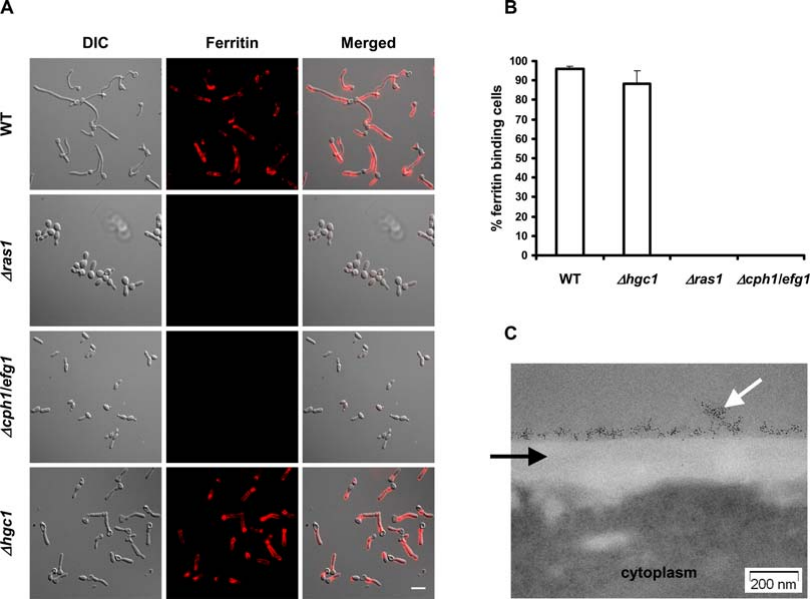
Ferritin binding of *C. albicans* requires hyphal formation. (A) *C. albicans* wild-type and mutant cells lacking key genes required for hyphal formation were incubated under hyphal-inducing conditions (RPMI 1640 medium, 37°C with 5% CO_2_) for 3 h. After 1 h in the presence of 100 µg/ml ferritin, cells were washed and ferritin was stained using immunofluorescence. Note that ferritin binding does not occur on the mother cell of hyphae. DIC, Differential Interference Contrast. Bar indicates 10 µm. (B) Quantification of *C. albicans* wild-type and mutant cells binding ferritin. For each strain the % ferritin binding cells is given for >100 randomly selected cells. The experiment was performed at least 3 times in duplicate. (C) Wild-type cells binding ferritin were analyzed under transmission electron microscopy. The black arrow points to the cell wall, the white arrow to ferritin molecules visualized by their electron density.

*C. albicans* cells precultured in iron limited medium (LIM0) were co-incubated with ferritin and then rinsed extensively. The ferritin that remained bound to the organisms was subsequently visualized with fluorescent labeled anti-ferritin antibodies. Hyphae of wild-type *C. albicans* bound ferritin whereas yeast-phase organisms did not (Figures 4A and 4B). The binding of ferritin to hyphae was also visualized by electron microscopy. Due to their high-electron density, ferritin molecules appeared as black particles in the electron micrograph adjacent to the fungal cell wall, indicating that ferritin bound to the cell surface, and not within the fungal cell wall (Figure 4C). *C. albicans* cells incubated under the same condition, but without ferritin, had no such electron dense particles on their surfaces (not shown).

The finding that ferritin was not bound by either yeast cells or the mother cells of hyphae suggested that ferritin binding was specific to *C. albicans* hyphae. To test this hypothesis further, we investigated the ferritin binding of *C. albicans Δras1* and *Δcph1/efg1* mutants that were unable to form hyphae, and did not express hyphal-specific genes [72],[73]. Both mutants were unable to bind ferritin (Figure 4A and 4B). Next, we tested the ferritin binding capacity of a *Δhgc1* mutant, which forms pseudohyphae rather than true hyphae, but still expresses hyphal-specific genes [74]. When grown under hyphal-inducing conditions (RPMI 1640, 37°C with 5% CO_2_), the *Δhgc1* mutant bound ferritin even though it did not form true hyphae (Figure 4A and 4B). These results suggest that the product of one or more hyphal specific genes is essential for *C. albicans* to bind ferritin.

To uncover which hyphal-associated activities are involved in ferritin binding, wild-type hyphae were killed with thimerosal or UV light and tested for ferritin binding. Cells killed with thimerosal still bound ferritin; whereas cells killed with UV light did not (Figure S3A). When untreated wild-type cells were mixed with 50% UV-killed cells, we observed 49.06%±4.27% ferritin binding. These data demonstrate that cell viability is not necessary for ferritin binding and that the ferritin receptor on the cell surface is inactivated by UV treatment. We also investigated whether iron availability influenced the extent of ferritin binding of wild-type hyphae. Cells grown under iron limiting conditions or in the presence of excess iron bound ferritin similarly (Figure S3B). Also, *C. albicans* hyphae were able to bind ferritin and apoferritin (a ferritin shell without iron) with similar efficiency (not shown) indicating that iron molecules within the ferritin shell were dispensable for binding of ferritin. Thus, these data indicated that the binding of ferritin by *C. albicans* is morphology associated, but not iron-regulated.

### Transcriptional Profiling of *C. albicans* Cells Binding Ferritin Identifies Putative Genes Necessary for Ferritin Binding

**Figure 5:**
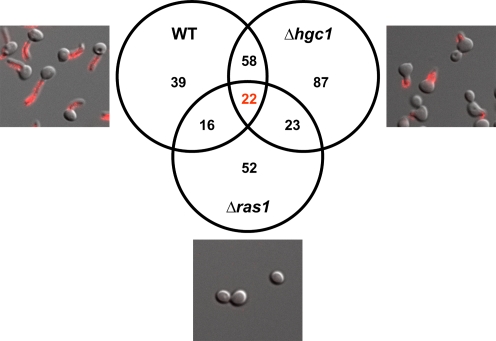
Transcription profiling identifies genes associated with ferritin binding. To identify genes necessary for ferritin binding, *C. albicans* wild-type, *Δhgc1* and *Δras1* mutant cells were incubated under conditions which favored ferritin binding to wild-type and *Δhgc1*, but not *Δras1* cells. RNA of each population of cells was isolated and used for microarray analysis. The micrographs show representative anti-ferritin labeled cells at the time point of RNA isolation. The Venn diagram indicates the number of genes up-regulated in wild-type and Δ*hgc1* and either unchanged or down-regulated in *Δras1*, as compared to a common control. Twenty two genes were up-regulated in wild-type and *Δhgc1*, but not *Δras1* cells as expected for a ferritin receptor. Microarray experiments were performed in four biological replicates (two of them using dye swap). Note that the schematic presentation of the Venn diagram combines up-regulated (wild-type and *Δhgc1*) and unaltered or down-regulated (*Δras1*) genes to clarify the selection strategy.

Transcriptional profiling was used to identify genes encoding putative ferritin receptors. We incubated a wild-type strain (true hyphae and ferritin binding), the *Δhgc1* mutant (yeast or pseudohyphae and ferritin binding) and *Δras1* (no hyphae and no ferritin binding) under hyphal-inducing conditions (RPMI medium, 37°C with 5% CO_2_) and in the presence of ferritin. After 1.5 h, the RNA from all three strains was isolated, labeled and hybridized to *C. albicans* microarrays. Microarray data from four independent experiments were analyzed. We reasoned that candidate genes encoding putative ferritin receptors should be up-regulated in wild-type and *Δhgc1* cells, but should be unaltered or down-regulated in the *Δras1* mutant (Figure 5). A total of 22 genes were identified with such an expression profile (Figure 5). Expression data shown in Table 2 indicate the genes that were up-regulated in wild-type cells but not in *Δras1* mutant cells. Three of these genes were known to encode hyphal-specific proteins that are cell surface localized as would be expected for a receptor protein. Consequently, these three genes were further investigated.

**Table 2:** Genes up-regulated in wild-type and *Δhgc1* cells, but unaltered or down-regulated in the *Δras1* mutant (Figure 5).

Gene name	Fold up-regulated in wild-type cells	Description
***ECE1***	22.0	cell elongation protein
*UME6*	6.6	transcription factor
*PUT2*	5.3	1-pyrroline-5-carboxylate dehydrogenase (by homology)
***ALS3***	5.0	agglutinin like protein
orf19.4805	5.0	unknown function
*FAS2*	4.4	fatty-acyl-CoA synthase (internal fragment)
*ADO1*	3.8	adenosine kinase (by homology)
orf19.2210	3.4	unknown function
*FAS1*	3.3	fatty-acyl-CoA synthase
*ABC1*	3.2	acyl-CoA binding (by homology)
*ACC1*	3.2	acetyl-CoA carboxylase (by homology)
*ERG25*	3.2	C-4 methylsterol oxidase (by homology)
orf19.4468	3.0	succinate dehydrogenase (by homology)
orf19.5147	2.9	unknown function
*UAP1*	2.8	UDP-N-acetylglucosamine pyrophosphorylase
orf19.801	2.8	unknown function
*FAS2*	2.7	fatty-acyl-CoA synthase (3-prime end)
***HYR1***	2.6	hyphally regulated protein
orf19.5126	2.6	unknown function
*RPS9B*	2.6	ribosomal protein (by homology)
orf19.2650.1	2.6	mitochondrial ribosomal protein (by homology)
orf19.1186	2.5	unknown function

Genes encoding hyphal surface proteins are in bold. Given data for wild-type cells were similar to data obtained for *Δhgc1* cells (not shown).

### Deletion of *ALS3* Precludes Ferritin Binding

The three genes encoding cell surface localized and hyphal-specific proteins were *ECE1*, *HYR1* and *ALS3*. *ECE1* (**E**xtent of **C**ell **E**longation) is a hyphal-specific gene with yet unknown functions. *ECE1* expression increases during elongation of the hyphal cell. This gene encodes a predicted cell membrane protein and the corresponding null mutant displays no obvious altered phenotype [40]. *HYR1* (**HY**phally **R**egulated) encodes a GPI-anchored protein that is predicted to be cell wall localized and is of unknown function [39]. Finally, *ALS3* (**A**gglutinin-**L**ike **S**equence) encodes a hyphal-specific cell wall protein which belongs to a family of adhesins (Als family) [75] and plays a crucial role in epithelial and endothelial adhesion and invasion [32].

**Figure 6:**
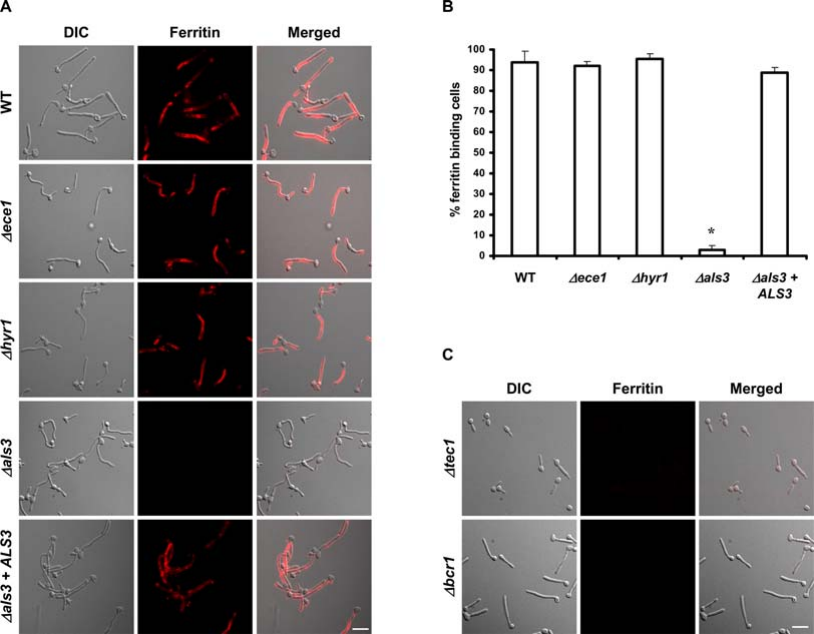
Als3 is essential for ferritin binding. (A) Mutants lacking either *ALS3*, *HYR1* or *ECE1*–the three selected genes predicted to encode ferritin receptors–were tested for ferritin binding. Bar indicates 10 µm. (B) Ferritin binding was quantified by counting >100 randomly selected cells using fluorescence microscopy. *, significant difference compared to wild-type (p<0.0001). (C) Ferritin binding by mutants lacking key regulators of *ALS3* expression (*Δtec1* and *Δbcr1*). Bar indicates 10 µm.

**Figure 7:**
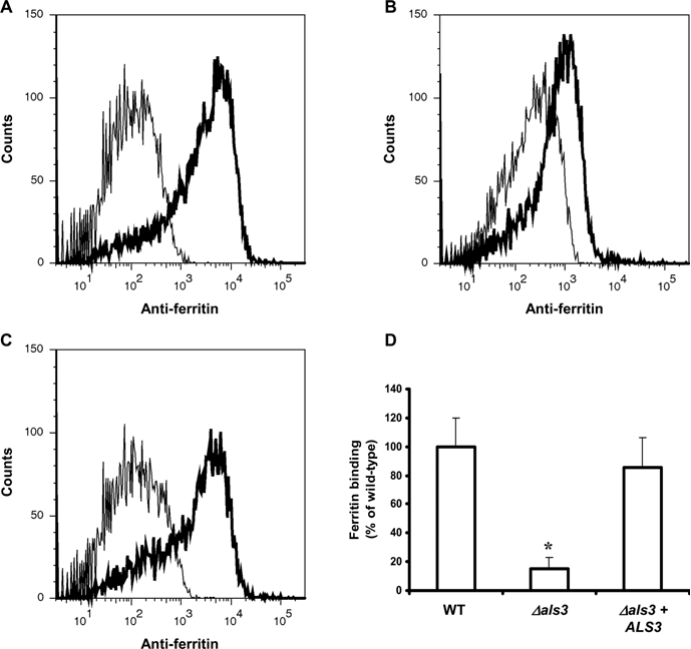
Flow cytometric detection of ferritin binding. *C. albicans* cells were incubated under hyphal-inducing conditions (RPMI, 37°C with 5% CO_2_) for 2 h. After 1 h in the presence of 100 µg/ml ferritin, cells were washed and ferritin was stained using indirect immunofluorescence and then analyzed using flow cytometry. (A) wild-type (CAF2-1); (B) *Δals3*; (C) *Δals3+ALS3*. Fluorescence data for 10,000 cells of each strain were collected. (D) Binding quantification. The data are expressed as a percentage of the results obtained with the wild-type strain (CAF2-1). The experiment was performed twice in duplicate. *, significant difference compared to wild-type (p<0.002).

The corresponding homozygous null mutants were tested for ferritin binding. Both, the *Δece1* and the *Δhyr1* mutants bound ferritin similarly to the wild-type strain (Figure 6A and B). In contrast, ferritin binding of the *Δals3* mutant was dramatically reduced (Figures 6 and 7). This defect in ferritin binding was restored when a wild-type copy of *ALS3* was reintegrated into the *Δals3* mutant (Figures 6 and 7).

These results suggested that Als3 plays a crucial role in ferritin binding and may in fact be the hyphal-specific ferritin receptor.

### Upstream Regulators of *ALS3* are Required for Ferritin Binding

If Als3 is a ferritin receptor, one would expect that mutants lacking factors that govern *ALS3* expression would also have an altered capacity to bind ferritin. Therefore, we tested two mutants that lacked *ALS3* transcriptional regulators. *BCR1* encodes a transcription factor which regulates the expression of certain hyphal-specific genes, including *ALS3* [76]. Furthermore, expression of *BCR1* itself depends on Tec1 [44]. Figure 6C shows that the presence of both transcriptional factors, Tec1 and Bcr1, is necessary for *C. albicans* cells to bind ferritin. These data reinforce the view that Als3 plays a key role in the capacity of *C. albicans* to bind ferritin.

### Binding is Necessary for Iron Acquisition from Ferritin

**Figure 8:**
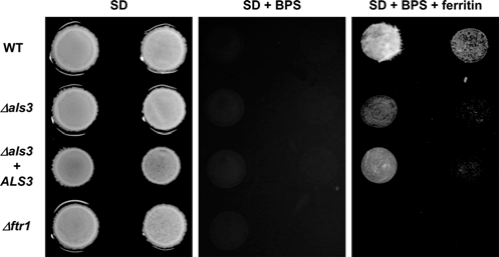
Binding is necessary for iron acquisition from ferritin. *C. albicans* wild-type (CAF2-1), *Δals3* and *Δftr1* were grown on media containing ferritin as the sole source of iron. SD agar was buffered using 100 mM HEPES (pH 7.4). BPS, iron chelator; ferritin, 2 µg/ml ferritin. Cells were spotted at two concentrations (left to right, 10^5^ and 10^4^ cells, respectively) for each strain. All plates were incubated for 3 days at 37°C under 5% CO_2_. The assay was performed three times.

To determine whether ferritin binding is necessary for the utilization of iron from this protein, we tested the growth of the *Δals3* mutant with ferritin as the sole iron source. The *Δals3* mutant grew very poorly on agar plates (pH 7.4) with ferritin as the sole source of iron (Figure 8). The reconstitution of one copy of the gene (*Δals3+ALS3* re-integrant strain), improved growth, although not to wild-type levels (Figure 8). Growth of the *Δals3* mutant in media with low iron content was not reduced, indicating that uptake of free iron is normal in the *Δals3* mutant (not shown). Therefore, Als3 is required for *C. albicans* hyphae to both bind and utilize ferritin as a source of iron.

Moreover, a mutant unable to form hyphae (*Δras1*) and thus unable to bind ferritin was also tested for growth on ferritin plates. As expected, *Δras1* displayed a reduced ability to grow with ferritin as the sole source of iron (Figure S4). This result reforces the key role of hyphal development and the hyphal associated expression of *ALS3* in the ability of *C. albicans* to obtain iron from ferritin.

### Als3 is a Ferritin Receptor

**Figure 9:**
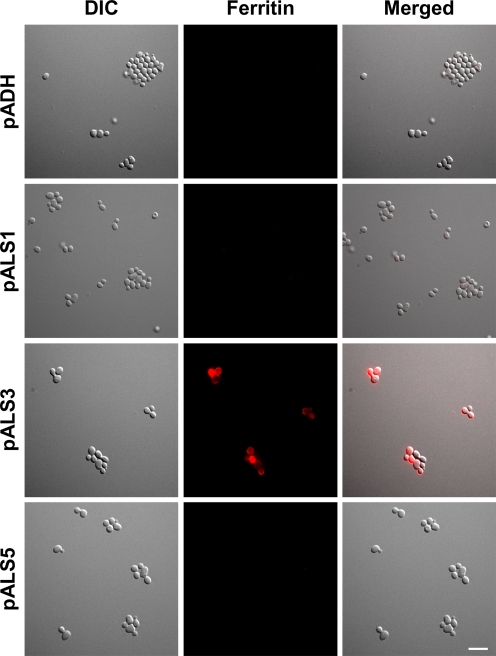
Als3 is a ferritin receptor. *S. cerevisiae* cells overexpressing *ALS1*, *ALS3*, *ALS5* (driven by the ADH promoter) or carrying an empty plasmid (pADH) were incubated for 15 min in the presence of 25 µg/ml ferritin coupled to a fluorescent dye. Cells were washed to remove non-bound ferritin and analyzed with fluorescence microscopy in duplicate repeated three times. Bar indicates 10 µm.

To elucidate whether Als3 itself can bind ferritin without an additional *C. albicans* surface factor, we tested the ferritin binding capacity of a strain of *S. cerevisiae* that expressed *C. albicans ALS3* [77]. Because *ALS3* is a member of a large gene family encoding similar proteins, we also analyzed two additional *S. cerevisiae* strains that expressed *ALS1* or *ALS5*, two closely related *ALS* genes. The strain that expressed *ALS3* strongly bound ferritin, whereas the strains that expressed *ALS1* or *ALS5* did not (Figure 9).

### Invading *C. albicans* Hyphae Bind Ferritin from Epithelial Cells during Infection

Next we investigated whether ferritin binding via Als3 occurs when *C. albicans* interacts with host cells. Oral epithelial cells were loaded with iron and then incubated with wild-type *C. albicans*, the *Δals3* mutant, or the *Δals3+ALS3* re-integrant strain for 6 h.

**Figure 10:**
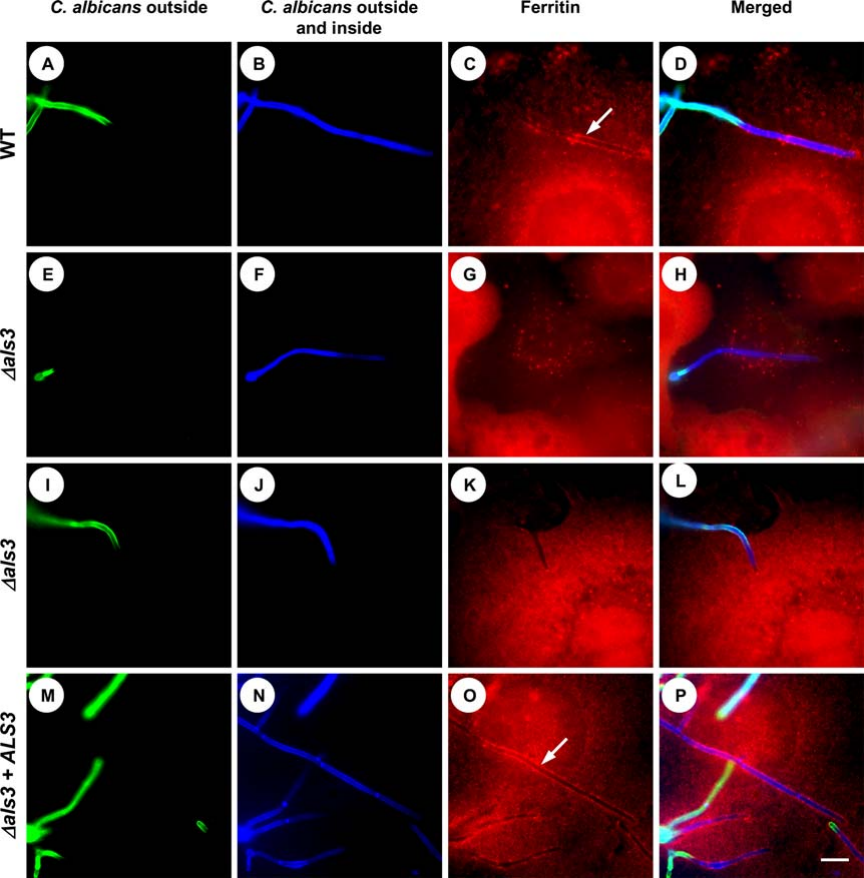
*C. albicans* hyphae invading oral epithelial cells bind ferritin. *C. albicans* wild-type (SC5314), *Δals3* mutant and *Δals3+ALS3* re-integrant cells were co-incubated with ferritin-enriched oral epithelial cells and differentially stained. (A), (E), (I) and (M); staining of extracellular (non-invaded) *C. albicans* with concanavalin A conjugated with fluorescein before cell permeabilization. (B), (F), (J) and (N); calcofluor white staining of whole *C. albicans* cells following epithelial cell permeabilization. (C), (G), (K) and (O); fluorescent dye (DY649) coupled antibody staining of ferritin. White arrows indicate hyphae surrounded by epithelial ferritin. (D), (H), (L) and (P); merged images. Bar in (P) indicates 10 µm.

To visualize ferritin molecules on cellular surfaces and to investigate whether the location of fungal cells had an influence on ferritin binding, we used an immunofluorescence approach with differential staining, which enabled us to discriminate between hyphae located on the epithelial cell surface and hyphae that had invaded into the epithelial cells (Figure 10 columns 1, 2 and 4). In addition, we used an anti-ferritin antibody to localize ferritin (Figure 10 column 3). As shown in Figure 10, hyphae of wild-type and *Δals3+ALS3* re-integrant strains invaded the epithelial cells and were surrounded by ferritin (white arrows in Figure 10). Very little ferritin accumulated around wild-type hyphae that had not invaded the epithelial cells (data not shown). In contrast, the few hyphae of the *Δals3* mutant that invaded the epithelial cells displayed no accumulation of ferritin (Figure 10G and K). These results indicate that *C. albicans* hyphae bind to ferritin in an Als3-dependent manner while invading epithelial cells.

### *C. albicans* Mutants Lacking Genes Essential for Iron Utilization from Ferritin are Unable to Damage Epithelial Cells

**Figure 11:**
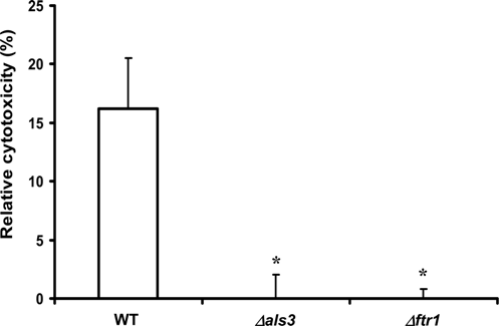
Iron uptake from ferritin plays a role in oral epithelial cell damage. *C. albicans* wild-type (CAF2-1), *Δals3* mutant and *Δftr1* mutant cells were co-incubated with oral epithelial cells. The monolayers were incubated for 8 h in serum-free RPMI 1640 with 10^6^ *C. albicans* cells and cell damage was quantified by monitoring the release of epithelial LDH into the medium. The experiment was performed five times in triplicate. *, significant difference compared to the wild-type (p<0.0001).

If binding to ferritin and utilizing host iron are important for *C. albicans* to cause an oral infection, one would expect that mutants lacking *ALS3* or *FTR1* would have a reduced potential to cause tissue damage as compared to wild-type cells. To test this prediction, we measured the extent of epithelial cell damage caused by wild-type, *Δals3* mutant and *Δftr1* mutant strains of *C. albicans*. We found that the *Δals3* and *Δftr1* mutants lost their capacity to damage epithelial cells (Figure 11). In contrast to *Δals3* mutant cells, which displayed strongly reduced invasion abilities when co-incubated with epithelial cells for 3 hours (not shown), *Δftr1* mutant cells showed the same invasion rate than the wild-type strain (Figure S5). Although hyphae of this mutant seemed shorter than the wild-type hyphae, there was no morphological differences between *Δftr1* mutant cells on epithelial cells and in RPMI medium alone (control) (not shown).

## Discussion

Iron availability is a critical factor for all pathogenic microbes and iron excess can accelerate pathogenicity [1], [78]–[80]. We observed that oral epithelial cells enriched in intracellular ferritin were more susceptible to tissue damage by wild-type *C. albicans* and that epithelial cells depleted of ferritin were significantly protected from damage. The reduced damage of iron depleted epithelial cells correlated with reduced invasion of *C. albicans*. It is possible that the treatment with the iron chelator affected both the host cells and the pathogen. Iron depleted epithelial cells may have a reduced ability to internalize fungal cells and limited accessibility of iron may reduce the capacity of *C. albicans* to both invade and damage epithelial cells. This model is supported by previous data. For example, endothelial cells incubated with an iron chelator before *C. albicans* infection were protected from injury by *C. albicans* [81] and the anti *Candida* activity of ciclopiroxolamine, a potent antifungal agent, is proposed to be mediated by iron chelation [82]–[84]. In contrast, when epithelial cells were loaded with exogenous iron, epithelial cell uptake of *C. albicans* was not affected. However, the increased iron reservoir was likely exploited by *C. albicans*, leading to increased epithelial cell damage. This increased damage was probably due to an enhanced production of virulence determinants (e.g. hydrolases) and hyphal extention. Therefore, it can be concluded that access to iron has a direct influence on the pathogenicity of *C. albicans*, probably by acting on both the host and the fungus. Furthermore, our data suggest that *C. albicans* is able to directly use ferritin as a source of iron.

It is known that ferritin is an extremely robust and resistant protein. Prior to this study, the only microorganism that has been known to exploit holoferritin as an iron source during interaction with host cells is *N. meningitidis* [21],[22]. However, this bacterium is not able to directly utilize iron from ferritin. Instead, it induces degradation of cytosolic ferritin by manipulating the host cellular machinery and thereby utilizes the resultant free cytosolic iron. To our knowledge, no published studies have so far demonstrated direct use of iron from host ferritin.

Nevertheless, a number of studies have suggested that certain microbial pathogens can use ferritin as an iron source during *in vitro* growth. For example, *Yersinia pestis* can grow on agar containing hemin, myoglobin, hemoglobin or ferritin [85]. A siderophore produced by *M. tuberculosis* (exochelin) can sequester iron from transferrin, lactoferrin and to a lesser extent from ferritin [86]. *L. monocytogenes* and *Burkholderia cenocepacia* can grow in liquid medium with ferritin as the sole source of iron [87],[88]. However, the microbial mechanisms of iron acquisition from ferritin are unknown and it is not clear whether ferritin from host cells can be used by any of these species. Furthermore, although ferritin seems to be almost indestructible under physiological conditions, iron may be released from ferritin *in vitro*, especially under condition of low pH. In our hands, even *S. cerevisiae* was able to utilize iron from ferritin under such conditions. Therefore, it is possible that previous observations of the microbial usage of ferritin *in vitro* were the results of non-physiological conditions.

In contrast to *S. cerevisiae*, *C. albicans* can use ferritin as the sole source of iron *in vitro* even when the growth medium was buffered at a physiological pH. Which mechanisms and activities are involved in iron acquisition from ferritin?

One possibility is that ferritin is degraded by extracellular proteolytic activity since it is known that *C. albicans* can secrete a family of aspartic proteases (Saps) with very broad substrate specificity [89]. However, it appears that extracellular degradation due to fungal proteases is not necessary for growth with ferritin, since mutants lacking the protease genes *SAP1-3* or *SAP4-6* were still able to grow on such medium. Indeed, an earlier study by Rüchel demonstrated that ferritin was the only tested protein which was resistant to proteolysis by Sap2, one of the major secreted proteases of *C. albicans* with an extremely broad substrate specificity [90], supporting the view that proteases are not involved in the ability of *C. albicans* to utilize iron from ferritin.

Since even *S. cerevisiae* was able to grow with ferritin when the pH of the medium was low (pH 5.0), we reasoned that the pH plays a crucial role in the release of iron from ferritin. It is known that ferritin is unstable at acidic pH [18] and that the natural recycling of iron from ferritin occurs in the acidic environment of lysosomes [19],[20]. Thus, it may be possible that *C. albicans* actively lowers the pH in its proximate vicinity. In fact, *C. albicans* was able to lower the pH of the medium during growth even on buffered ferritin plates (Figure S2). Additionally, the fungus was only able to use ferritin as an iron source under conditions which allowed acid production (glucose, but not casamino acids as a carbon source) and acidification of the surrounding environment (low concentrations of buffer at pH 7.4). Similarly, it has been observed that the bacterial pathogen *Staphylococcus aureus*, under iron starvation, decreases the local pH resulting in the release of iron from transferrin [91].

It is also possible that *C. albicans* can produce and secrete reductants, which are able to sequester iron from ferritin. Such a process would indeed be favoured by acidification of the surrounding media. In agreement with this model, reductants or chelators such as thioglycolic acid, ascorbate, and aceto- and benzohydroxamic acids are capable of releasing iron from the ferritin core [92]–[94]. Underscoring the importance of pH in the release of iron from ferritin, this process is increased at pH 5.2 in comparison to pH 7.4 [94]. However, since we demonstrated that binding is necessary for ferritin iron exploitation by *C. albicans*, it can be hypothesized that a surface factor rather than a secreted factor is necessary for ferric iron reduction from the ferritin core. Another possible speculation is that reductases on the *C. albicans* cell surface can reduce ferric iron from the ferritin core and that this process may be facilitate under acidic pH.

Although we do not have experimental evidence that local acidification occurs *in vivo* during infection, transcriptional profiling of *C. albicans* during experimental infections suggests that the local environment of at least some cells in fact changes from neutral to acidic pH during invasion and tissue damage. For example, we have found that the acid induced gene, *PHR2* is up-regulated during tissue invasion [34].

In addition to the ability to acidify the environment, *C. albicans* requires the reductive high-affinity iron uptake pathway to exploit iron from ferritin. Mutants lacking either the high-affinity permease Ftr1 or the copper transporter Ccc2 (which is essential for the reductive pathway) [63],[64] did not grow on ferritin plates even when the initial pH was low. Therefore, we conclude that a combination of active acidification and uptake via the high-affinity permease are key mechanisms in this process. As a third prerequisite, we hypothesized, that a close association between *C. albicans* cells and ferritin is required for the release of iron from ferritin and subsequent uptake into the fungal cell. This close contact is facilitated by binding of ferritin on the fungal surface.

In principle it may also be postulated that a yet unknown molecule is secreted by *C. albicans*, which binds ferritin and subsequently delivers the iron protein to a surface receptor, similar to some bacteria which can secrete haemophores that bind extracellular haemoglobin and mediate its delivery to surface receptors [95]. However, such a mechanism is unlikely to be involved in ferritin-binding by *C. albicans* since fungal cells that were killed with thimerosal and then washed, removing any secreted factors, were still able to bind ferritin.

Interestingly, fungal cells killed via exposure to UV-light lost their ability to bind ferritin. This result suggests that ferritin-binding at the cell surface is mediated by a receptor which is inactivated by UV treatment. In support of this possibility, it is known that certain proteins can be inactivated by exposure to UV light [96].

Several lines of evidence suggest that the cell surface protein, Als3 is a receptor that binds ferritin and facilitates iron acquisition from this protein. (1) Only hyphae, but not yeast cells bound ferritin and Als3 is known to be a hyphal-specific protein. However, the binding of ferritin did not need the hyphal morphology, since a mutant lacking Hgc1 [74] did not produce true hyphae, but still bound ferritin (Figures 4A and 4B) and expressed *ALS3* (not shown). (2) Mutants lacking transcription factors known to regulate *ALS3* expression (Tec1, Bcr1) [44],[45] had a reduced ability to bind ferritin. In agreement with this, a mutant that was unable to form hyphae (*Δras1*) and that did not express *ALS3*, also displayed reduced binding of ferritin and reduced growth on ferritin plates. (3) A mutant lacking *ALS3* was dramatically reduced in its ability to bind ferritin and displayed poor growth on ferritin plates. The *Δals3+ALS3* re-integrant strain had a restored ability to bind ferritin and a partially restored ability to grow on ferritin plates, although not to wild-type levels, possibly due to a gene dosage effect. Finally, (4) a *S. cerevisiae* strain expressing Als3 was able to bind ferritin.

Binding of ferritin to hyphal surfaces was observed with both exogenously added purified ferritin and during the interaction of *C. albicans* with intact epithelial cells. Only hyphae, but not yeast cells showed bound ferritin during interaction with epithelial cells. Furthermore, ferritin accumulation was predominantly observed on those hyphae that had invaded the epithelial cells. Finally, the hyphae of the *Δals3* mutant did not show ferritin accumulation.

**Figure 12:**
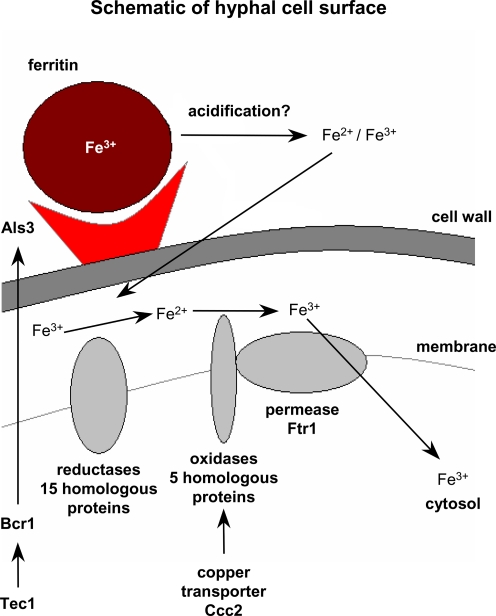
Proposed model for iron utilization from ferritin by *C. albicans*. Ferritin is a novel iron source used by *C. albicans*. In its hyphal form, *C. albicans* binds ferritin using Als3. Acidification of the surrounding environment mediates iron release from the ferritin shell and the released iron is then transported into the cell via the reductive pathway.

Taken together, these data suggest that ferritin can be used as an iron source by *C. albicans* via direct binding by Als3 on the surface of hyphae, iron release is then mediated by acidification and uptake is facilitated by the reductive pathway (Figure 12).

Although we do not have direct evidence that ferritin is in fact used as an iron source during interaction with epithelial cells, these data at least suggest that ferritin is in close contact to invading *C. albicans* hyphae and thus may be exploited by the above proposed mechanism. This view is supported by the fact that both the *Δals3* mutant and the *Δftr1* mutant completely lost their capacities to damage epithelial cells *in vitro*. Furthermore, the *Δals3* mutant has significantly reduced capacity to damage epithelial cells in the reconstituted human epithelium model [97]. However, it should be noted that this reduced damage is likely due to a combination of reduced adherence [97], reduced invasion [32], and reduced ability to use ferritin as an iron source. Interestingly, hyphae of the *Δftr1* mutant displayed the same invasion rate than the wild-type strain, suggesting that this mutant can initially invade the epithelial cells, but is not able to damage host cells possibly because it can not use ferritin as an intracellular available iron source.

Several studies have shown that pathogenic microbes link the availability of iron with virulence attributes. In this study, we show that a similar link between the regulation of an iron acquisition system and virulence attributes exists in *C. albicans*. In fact, the regulation of the ferritin receptor Als3 is independent from external iron sources and seems to be strictly linked to hyphal formation, one of the most extensively investigated virulence attributes of *C. albicans* [30],[98].

Therefore, iron acquisition of the intracellular iron storage protein ferritin is hyphal regulated. Hyphal formation is also associated with adhesion, proteolytic activity, cellular invasion and damage [32]–[34],[89],[99], and the hyphal form of the organism is the predominant morphology that reaches the intracellular compartments of epithelial cells where ferritin is located. Therefore, *C. albicans* co-regulates morphology, invasion, tissue damage and an iron acquisition system. This view may explain why iron acquisition from ferritin is a hyphal-specific property and does not occur with the normally non-invasive yeast cells.

A second potential link exists between the external pH, hyphal formation and iron acquisition. It is well known that the external pH influences hyphal formation [100],[101] and we recently reported that pH-dependent hyphal formation is crucial for liver invasion [34]. During liver invasion *C. albicans* cells are exposed to a neutral or alkaline pH and iron limited conditions as reflected by transcriptional profiles [34]. Availability of iron for fungal cells within a human host is even more difficult in neutral or slight alkaline pH conditions such as those found in the liver tissue (pH 7.4) because the balance between the soluble Fe^2+^ ion and the insoluble ferric form Fe^3+^ shifts towards the insoluble form [41]. Therefore, the formation of hyphae and expression of Als3 in response to neutral pH may facilitate iron acquisition by *C. albicans*.

Interestingly, the expression of *ALS3* is not absolutely linked to the hyphal morphology in wild-type cells. Sosinska *et al.* [102] recently observed that hypoxic conditions and iron restriction in a vagina-simulative medium affected cell morphology and the cell wall proteome of *C. albicans*. One of the proteins found in yeast cells under these iron limited conditions was Als3, which indicates that even proteins which are strictly hyphal-associated under most growth conditions, may be expressed in the yeast form. Similarly, White and co-workers recently showed that *C. albicans* expresses a number of hyphal-specific genes (such as *ECE1*) in a murine gut model of commensalism, whilst growing in the yeast morphology [103]. The observation that yeast cells express Als3 under iron limited conditions may further support the view that this protein is involved in iron acquisition from the host. However, the expression of *ALS3* is not directly linked to low iron conditions since two studies that analyzed the influence of iron on the genome wide gene expression of *C. albicans* [47],[83] found that iron starvation did not increase the expression of *ALS3*.

The Als protein family of *C. albicans* encodes large cell-surface GPI-glycoproteins that were originally implicated in the process of adhesion to host surfaces [75],[104]. Expression of Als3, was shown to be hyphal-specific [36] and was observed *in vivo* during oral and systemic infection [33],[34]. In addition to its adhesion properties, Als3 was recently shown to be an invasin that binds to cadherins and induces endocytosis by host cells [32]. In this study, we made the intriguing observation that Als3 has a third function in iron acquisition by binding to host ferritin, indicating that this single member of a protein family has multiple virulence-associated functions.

## Materials and Methods

### Fungal growth conditions and strains

*C. albicans* were grown in liquid YPD medium (1% yeast extract [Merck, http://www.merck.de], 2% bactopeptone [Difco, http://www.bdbiosciences.com], and 2% D-glucose [Roth, http://www.carl-roth.de]) in a shaking incubator at 30°C for 8 h. Subsequently, the cultures were diluted 1∶1000 in LIM0 medium [105] and incubated in a shaking incubator at 30°C overnight for iron starvation. For non-starved cells, precultures were incubated in YPD medium overnight at 30°C with shaking. The yeast cells were harvested by centrifugation, washed three times in filter sterilized ultra-pure water and counted using a hemacytometer. Strains of *C. albicans* and *S. cerevisiae* used in this study are listed in Table 3 and Table 4, respectively.

**Table 3:** *C. albicans* strains used in this study.

Strain	Genotype	Reference
SC5314	wild-type	[110]
CAF2-1	*ura3::imm434*/*URA3*	[111]
CAI-4+CIp10	*ura3*::*imm434*/*ura3*::*imm434*+CIp10 (*URA3*)	[108]
*Δals3*	*ura3*::*imm434::URA3-IRO1*/*ura3*::*imm434 arg4*::*his*G/*arg4*::*his*G *his1*::*his*G/*his1*::*his*G *als3::ARG4/als3::HIS1*	[76]
*Δals3+ALS3*	*ura3*::*imm434::URA3-IRO1*/*ura3*::*imm434 arg4*::*his*G/*arg4*::*his*G *his1*::*his*G/*his1*::*his*G *als3::ARG4::ALS3/als3::HIS1*	[76]
*Δbcr1*	*ura3*::*imm434*/*ura3*::*imm434 arg4*::*his*G/*arg4*::*his*G *his1*::*his*G/*his1*::*his*G::pHIS1 *bcr1::ARG4/bcr1::URA3*	[44]
*Δccc2*	*ura3::imm434/ura3::imm434 ccc2::hisG/ccc2::hisG-URA3-hisG*	[63]
*Δccc2+CCC2*	*ura3::imm434/ura3::imm434 ccc2::hisG/ccc2::URA3::CCC2*	[63]
*Δcph1/efg1*	*ura3::imm434/ura3::imm434 cph1::hisG/cph1::hisG efg1::hisG/efg1::hisG-URA3-hisG*	[73]
*Δece1*	*ura3::imm434::URA3-RO1/ura3::imm434 ece1::hisG/ece1::hisG*	[40]
*Δftr1*	*ura3::imm434/ura3::imm434 ftr1::hisG/ftr1::hisG-URA3-hisG*	[64]
*Δftr1+FTR1*	*ura3::imm434/ura3::imm434 ftr1::hisG/ftr1::URA3::FTR1*	[64]
*Δhgc1*	*ura3::imm434/ura3::imm434 arg4::hisG/arg4::hisG his1::hisG/his1::hisG hgc1::ARG4/hgc1::HIS1*	[74]
*Δhgc1+CIp10*	*ura3::imm434/ura3::imm434 arg4::hisG/arg4::hisG his1::hisG/his1::hisG hgc1::ARG4/hgc1::HIS1*+CIp10 (*URA3*)	This study
*Δhyr1*	*ura3::imm434/ura3::imm434 hyr1::hisG/hyr1::hisG-URA3-hisG*	[39]
*Δras1*	*ura3::imm434/ura3::imm434 ras1::hisG/ras1::hph*	[72]
*Δras1+CIp10*	*ura3::imm434/ura3::imm434 ras1::hisG/ras1::hph+*CIp10 (*URA3*)	This study
*Δrbt5*	*ura3::imm434/ura3::imm434 rbt5::hisG/rbt5::hisG-URA3-hisG*	[13]
*Δsap1-3*	*ura3::imm434/ura3::imm434 sap1::hisG/sap1::hisG sap2::hisG/sap2::hisG sap3::hisG/sap3::hisG-URA3-hisG*	[112]
*Δsap4-6*	*ura3::imm434/ura3::imm434 sap4::hisG/sap4::hisG sap5::hisG/sap5::hisG sap6::hisG/sap6::hisG-URA3-hisG*	[37]
*Δsit1*	*ura3::imm434/ura3::imm434 sit1::hisG/sit1::hisG-URA3-hisG*	[50]
*Δtec1*	*ura3::imm434 /ura3::imm434 tec1::hisG/tec1::hisG+*pVEC (*URA3*)	[45]

**Table 4:** *S. cerevisiae* strains used in this study.

Strain	Genotype	Reference
ATCC9763	wild-type	American Type Culture Collection
pADH1	*leu2 his3 trp1 ura3*+pADH1	[77]
pALS1	*leu2 his3 trp1 ura3*+pALS1	[77]
pALS3	*leu2 his3 trp1 ura3*+pALS3	[77]
pALS5	*leu2 his3 trp1 ura3*+pALS5	[77]

### Oral epithelial cells

The epithelial cell line TR146, derived from a squamous cell carcinoma of buccal mucosa [106], was kindly provided by Cancer Research Technology (http://www.cancertechnology.co.uk). TR146 cells were routinely grown in RPMI 1640 medium (PAA, http://www.paa.com) supplemented with 10% fetal bovine serum (FBS; PAA). For experiments, epithelial cells were used between passages 10 to 20. Monolayers with 70–90% confluent cells in 24 well plates were additionally incubated for 24 h in three different conditions: (1) RPMI 1640 with 50 µM bathophenanthrolinedisulfonic acid disodium salt (BPS; iron chelator; Sigma-Aldrich, http://www.sigmaaldrich.com); (2) RPMI 1640 with 10% FBS; (3) RPMI 1640 with 10% FBS and indicated concentrations of iron chloride (FeCl_3_; Merck). After 24 h incubation, monolayers were washed twice with phosphate-buffered saline without calcium or magnesium (PBS) and serum-free RPMI 1640 medium was added. Each well was infected with ∼10^6^ *C. albicans* cells and incubated for 8 h. Supernatants were removed for LDH measurements. All incubations were performed in a humidified incubator at 37°C in 5% CO_2_.

To monitor the ferritin content of cells, the uninfected monolayers were fixed with Roti®-Histofix 4% (Roth) and the ferritin content of the cells was visualized under the microscope using immunofluorescence. Briefly, fixed monolayers were permeabilized through incubation with 0.1% Triton X-100 (Serva, http://www.serva.de) for 15 min at room temperature and washed three times with PBS. Next, the samples were blocked using Image-iT™ FX signal enhancer (Invitrogen, http://probes.invitrogen.com/products/) for 30 min at room temperature in a humidity chamber. Cells were again washed three times with PBS and incubated with rabbit anti-ferritin antibody (Sigma-Aldrich) coupled with dye DY-649 (Dyomics, http://www.dyomics.com) diluted 1∶1000 in PBS with 1% bovine serum albumin (BSA, Sigma-Aldrich) for 1 h at room temperature. Finally, cover-slips were washed three times with PBS, inverted and mounted on a microscope slide with ProLong® Gold Antifade Reagent with 4′,6-diamidino-2-phenylindole dihydrochloride (DAPI) (Invitrogen). The samples were analyzed in duplicates using a Leica DM 5500B microscope (Leica, http://www.leica-microsystems.com). The same exposure time and light intensity were used to analyze all samples, permiting comparisons. For every sample, 10 randomly chosen fields per cover-slip were photographed using a DFC 350 FX camera (Leica). A representative picture of each condition was selected.

### Epithelial cell monolayer damage assay

Epithelial cell damage caused by *C. albicans*, was determined by the release of lactate dehydrogenase (LDH) into the medium using a Cytotoxicity Detection Kit–LDH (Roche, http://www.roche.de). The assays were performed according to the manufacturer instructions and the measurements were performed in duplicates.

To measure epithelial cell damage, the following calculation was used: 100×(ECa−C1−C2)/(100L−C1) = relative cytotoxicity (%). Absorbance measured at OD 490–600 directly correlates with LDH activity. ECa = epithelial cells infected with *C. albicans*; C1 = control 1–uninfected epithelial cells; C2 = control 2–only *C. albicans*; 100L = 100% lysis (0.2% Triton-X 100, Serva). Controls 1, 2 and 100% lysis were determined individually for each treatment.

### Ferritin agar plates

To investigate whether *C. albicans* was able to grow with ferritin as the sole source of iron, we added 350 µM BPS to the SD agar medium (6.7 g/l yeast nitrogen base, YNB [Difco]; 20 g/l D-glucose; 20 g/l purified agar [Oxoid, http://www.oxoid.com]). Additionally, HEPES buffer (Sigma-Aldrich) was added to the medium as indicated and the pH was adjust to 7.4 using a 5 M NaOH stock solution (Roth). To prevent active acidification of the medium by the fungus, 20 g/l casamino acids (Difco) was used in place of D-glucose. The ferritin solution (ferritin from horse spleen [Sigma-Aldrich]) was diluted 1∶100 in a dilution buffer (5 mM HEPES; 0,1 M NaCl [Roth]) and passed through a Microcon YM-100 Centrifugal Filter Unit (Millipore, http://www.millipore.com). The retentate was collected in a fresh 1.5 ml microcentrifuge-tube and the original volume was adjusted with the dilution buffer. Afterwards, this ferritin solution was plated out on agar surfaces at indicated concentrations. To monitor the pH changes in the medium during *C. albicans* growth, the pH indicator bromocresol green (Sigma-Aldrich) was added to the medium at a concentration of 3.9 mg/l.

### Ferritin binding assay

*C. albicans* cells growing under iron limitation, as described above, were washed and enumerated. Approximately 5×10^5^ cells were added per well in a 24 well plate (TPP, http://www.tissue-cell-culture.com) containing Poly-L-Lysine-coated (Biochrom AG, http://www.biochrom.de) 12-mm diameter glass cover-slips and 1 ml RPMI 1640. The cells were incubated for 3 h at 37°C under 5% CO_2_ to induce hyphae. Afterwards, the cells were washed once with PBS and incubated for 1 h in 1 ml PBS with 1% bovine serum albumin (BSA) and 100 µg/ml ferritin. Subsequently, the cells were washed three times with PBS to remove non-bound ferritin and fixed with 500 µl Roti®-Histofix 4%.

To test if viability is necessary for ferritin binding, *C. albicans* hyphae (3 h in RPMI 1640 at 37°C and 5% CO_2_) were killed using two different approaches: either 1.5 h incubation at room temperature with 0.05% Thimerosal (Sigma-Aldrich) or 2 times exposition to 0.5 J/cm^2^ UV light in a UV-crosslinker with a 254 nm low pressure mercury-vapor lamp (Vilber-Loumart, http://www.vilber.de). Complete killing without residual viability of cells was checked by plating the cells on YPD agar plates. After killing, the cells were incubated with ferritin and fixed as described above.

The fixed cells were washed three times with PBS and incubated with rabbit anti-ferritin antibody coupled with dye DY-649 diluted 1∶2000 in PBS with 1% BSA for 1 h at room temperature. Next, the cover-slips were inverted and mounted on a microscope slide with ProLong® Gold Antifade Reagent (Invitrogen) and cells were visualized using a Leica DM 5500B microscope (Leica). Photomicrographs were taken using a DFC 350 FX camera (Leica). To quantify how many *C. albicans* cells bound ferritin, at least 100 cells per cover-slip were counted and percent binding was calculated by counting the total number of cells and the number of cells displaying fluorescent signal. All binding assays were performed in duplicates. Cells incubated without ferritin were used as a negative control.

Because *S. cerevisiae* cells were detached during the washing steps described above, a different approach was used. The use of a fluorophore-coupled ferritin reduced the number of washing steps in the staining procedure and consequently left more cells on the coverslip for observation by fluorescent microscopy. Briefly, 5×10^5^ cells were added per well in a 24 well plate containing Poly-L-Lysine-coated 12-mm diameter glass cover-slips in 1 ml RPMI 1640. The cells were incubated for 1 h at 30°C. Afterwards, the medium was removed and 250 µl PBS with 1% BSA and 25 µg/ml ferritin coupled with dye DY-649 was added. After 15 min at 30°C, the cells were washed once with PBS, fixed, mounted and visualized under the microscope as described above for *C. albicans* cells.

### Transmission electron microscopy

*C. albicans* wild-type cells (SC5314) were grown on poly-L-lysine-coated cover-slips (0.5 mm in diameter) in the presence or absence of 100 µg/ml ferritin for 6 h in RPMI 1640. Afterwards, the cells were washed with PBS four times to remove non-bound ferritin and then immersed in fixative (4% formaldehyde, prepared from para-formaldehyde [Roth] and 0.1% glutaraldehyde [Roth] in 0.05 M HEPES ) at room temperature. After three min the fixative was replaced with fresh fixative and stored at 4°C overnight. The samples were dehydrated in ethanol (Roth) by progressively lowering the temperature to −35°C and infiltrated with Lowicryl K4M resin (Polysciences, http://www.polysciences.com) at −35°C [107]. The resin polymerization was carried out under UV light at −35°C for 24 h and for 10 h at 0°C. Ultra thin sections (60–80 nm thick) were produced with an Ultracut S (Leica) and a diamond knife. Sections were collected on formvar filmed copper slot grids. Bright-field transmission electron microscopy was performed with an EM902 (ZEISS, http://www.zeiss.de) at 80 kV. Images were recorded with a 1 k CCD camera (Proscan, http://www.proscan.de).

### Flow cytometry analysis of ferritin binding

Flow cytometry was used to quantify the binding of ferritin on the surface of *C. albicans* hyphal cells. *C. albicans* cells were grown under iron limitation, as described above, washed and counted. Approximately 10^6^ cells in 1 ml RPMI 1640 medium were added to poly-L-lysine-coated (Biochrom) 12-mm diameter glass cover-slips in a 24 well tissue-culture plate (TPP). The cells were incubated for 2 h at 37°C in 5% CO_2_ to induce hyphae. Next, the cells were washed once with PBS and incubated for 1 h in 0.5 ml PBS with 1% bovine serum albumin (BSA) and 100 µg/ml ferritin. Subsequently, the cells were washed three times with PBS to remove non-bound ferritin and fixed with 500 µl Roti®-Histofix 4%. The fixed cells were washed three times with PBS and incubated with rabbit anti-ferritin antibody (Sigma-Aldrich) diluted 1∶500 in PBS with 1% BSA for 1 h at room temperature. After washing, the cells were incubated with a goat anti-rabbit secondary antibody conjugated with Alexa 488 (Invitrogen) diluted 1∶500. Finally, the cells were detached from the cover-slips using a pipet point and resuspended in 0.5 ml PBS. The fluorescent intensity of the hyphae was measured using a LSRII flow cytometer (Becton Dickinson, http://www.bd.com). Fluorescence data for 10,000 cells of each strain were collected.

### Immunofluorescence of infected epithelial cells

Ferritin enriched epithelial cell monolayers (described above) were washed twice with PBS and infected with ∼10^5^ *C. albicans* cells in serum-free RPMI 1640 medium for 6 h. Next, the samples were washed twice with PBS and fixed with 500 µl Roti®-Histofix 4%. *C. albicans* cells and TR146 cells were incubated separately and used as controls. All incubation times were performed in a humidified incubator at 37°C in 5% CO_2_.

To stain *C. albicans* cells localized only outside epithelial cells, before permeabilization, the samples were incubated with 12.5 µg Concanavalin A–fluorescein conjugate (Invitrogen) in PBS for 45 min at room temperature. After washing, the cells were permeabilized by incubation with 0.1% Triton X-100 for 15 min at room temperature. After washing three times with PBS, the samples were blocked using Image-iT™ FX signal enhancer (Invitrogen) for 30 min at room temperature in a humidity chamber. After washing three times with PBS, the cells were incubated with rabbit anti-ferritin antibody coupled with dye DY-649 diluted 1∶1000 in PBS with 1% BSA for 1 h at room temperature. To stain *C. albicans* cells localized outside and inside epithelial cells, the samples were incubated with 10 µg/ml Calcofluor White (Sigma) in 0,1 M Tris-hydrochloride (pH 9.0 [Roth]) for 20 min at room temperature.

Finally, cover-slips were washed three times with ultra pure water, inverted and mounted on a microscope slide with ProLong® Gold Antifade Reagent. At least two experiments in duplicates were analyzed using a Leica microscope and 10 randomly chosen fields per cover-slip were photographed. A representative picture of each strain was selected.

### Invasion of ferritin depleted or enriched oral epithelial cells

Ferritin depleted or enriched oral epithelial cell monolayers (as described above) were washed twice with PBS and infected with ∼10^5^ iron starved *C. albicans* cells in serum-free RPMI 1640 medium for 3 h. The samples were washed twice with PBS and fixed with 500 µl Roti®-Histofix 4%. *C. albicans* cells alone were incubated separately and used as control. All samples were incubated in a humidified incubator at 37°C and 5% CO_2_. The samples were stained to distinguish invading from non-invading fungal cells as described above. At least 100 randomly selected organisms were analyzed and the percentage of organisms that had invaded the epithelial cells was calculated.

### Sample preparation for RNA extraction

*C. albicans* cells growing under iron limitation, as described above, were washed and enumerated. Approximately 2×10^6^ cells were added per well in a 24 well plate containing Poly-L-Lysine -coated 12-mm diameter glass cover-slips in 1 ml RPMI 1640 with 100 µg/ml ferritin. The strains used were CAI4 carrying CIp10; *Δhgc1* carrying CIp10 and *Δras1* carrying CIp10. The plasmid CIp10 was used to reconstitute *URA3* into the RP10 locus of each strain [108]. After 1.5 h incubation at 37°C under 5% CO_2_, the medium was removed and 100 µl peqGOLD RNAPure (PeqLab, http://www.peqlab.de) was added per well. The cells were immediately removed from the cover-slips using a pipette point. For each strain, cells from 12 wells were pooled in a microcentrifuge tube and immediately shock frozen in liquid nitrogen. To verify that ferritin was bound to *C. albicans* hyphae as observed before, additional cover-slips for each strain were fixed and ferritin was stained as described.

#### RNA extraction and labeling

Frozen cells were lysed and homogenized (Precellys 24, PeqLab) with glass beads (0.5 mm, Roth). Total RNA was extracted as previously described [109]. Total RNA was linearly amplified and labeled using the ‘Low RNA Input Fluorescent Linear amplification Kit’ (Agilent Technologies, http://www.agilent.de).

### Microarray hybridization and analysis

For transcriptional profiling, *C. albicans* microarrays (Eurogentec) were used as previously described [109]. RNA was co-hybridized with a common reference (RNA from SC5314 grown in YPD medium, mid-log phase, 37°C). Slides were hybridized, washed and scanned as described [109]. Data normalization (LOWESS) and analysis were performed in Gene-Spring 7.2 software (Agilent Technologies). Reliable expression of genes was defined as normalized expression of present genes that did not vary more than 1.5 standard deviations within replicate arrays. Genes were defined as differentially expressed if their expression was at least 2 times stronger or 2 times weaker in at least one strain compared to the common reference. Using the Benjamini and Hochberg false discovery test, a *p*-value<0.05 was considered as significant. Microarray data from four independent experiments (two of them with dye swap) were used. To identify genes involved in ferritin binding, genes were selected that were up-regulated (≥2.5 increase in expression compared to the common reference) in wild-type and Δ*hgc1* cells, but unaltered or down-regulated (≤1.5 of the common reference expression) in the Δ*ras1* mutant. Raw data have been deposited in NCBIs Gene Expression Omnibus (GEO, http://www.ncbi.nlm.nih.gov/geo/) and are accessible through GEO series accession number GSE11490.

### Statistical Analysis

Statistical significances (p-values) were calculated with the Student's two-tailed t-test function in Microsoft Excel, with exception of the microarray analysis described above.

## Supporting Information

Figure S1 *C. albicans* can acidify the medium during growth on ferritin plates. *C. albicans* wild-type (SC5314) was grown on media containing ferritin as the sole source of iron and bromocresol green (3.9 mg/ml) as a pH indicator. SD agar was buffered using 25 mM HEPES (pH 7.4). BPS, iron chelator; ferritin, 15 µg/ml ferritin. All plates were incubated for 4 days at 37°C under 5% CO_2_. Blue indicates pH values higher than 5.5. Green indicates pH values between 5.5 and 4. Yellow indicates pH values below 4. The assay was performed twice in duplicate.
(6.36 MB TIF)

Figure S2 Examples of ferritin plates as described in Table 1. SD agar was buffered using 100 mM HEPES (pH 7.4). BPS, iron chelator. Ferritin, 5 µg/ml ferritin. All plates were incubated for 3 days at 37°C under 5% CO_2_.
(2.68 MB TIF)

Figure S3 Ferritin binding does not require live cells or iron limitation and is UV sensitive. (A) Comparison of ferritin binding between live and dead cells (using thimerosal or UV light). (B) Cells from iron limitation medium (LIM0) or from YPD were used for the ferritin binding assay. Additionally, cells from the same YPD preculture were tested for ferritin binding with the addition of 50 µM iron chloride during the binding assay.
(0.30 MB TIF)

Figure S4 Growth of selected mutants on ferritin plates. SD agar was buffered using 100 mM HEPES (pH 7.4). BPS, iron chelator. Ferritin, 5 µg/ml ferritin. All plates were incubated for 3 days at 37°C under 5% CO_2_.
(1.59 MB TIF)

Figure S5 Invasion of epithelial cells by *Δftr1*. Aproximately 10^5^ iron starved wild-type *C. albicans* cells (SC5314) or *Δftr1* mutant cells were co-incubated with epithelial cells for 3 h. After fixation the samples were differentially stained and analysed under the fluorescence microscope. The experiment was performed three times in duplicate. No significant difference was observed between the wild-type strain and the *Δftr1* mutant strain.
(0.30 MB TIF)
