# Supplementary figures and images for: The Hyphal-Associated Adhesin and Invasin Als3 of Candida albicans Mediates Iron Acquisition from Host Ferritin
Source: PLoS Pathog. 2008 Nov 21;4(11):e1000217. doi: 10.1371/journal.ppat.1000217 (PMC2581891; doi:10.1371/journal.ppat.1000217)

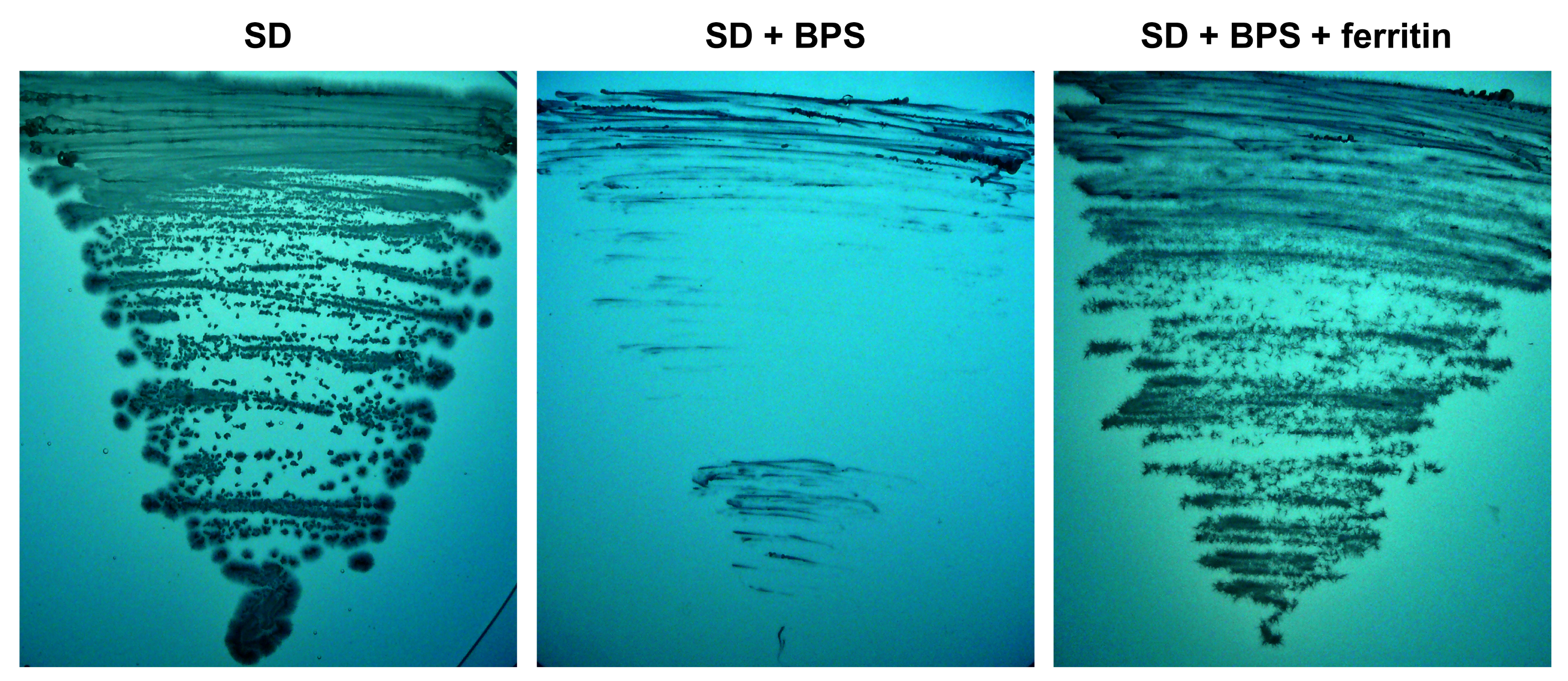

Supplement: Figure S1 — C. albicans can acidify the medium during growth on ferritin plates. C. albicans wild-type (SC5314) was grown on media containing ferritin as the sole source of iron and bromocresol green (3.9 mg/ml) as a pH indicator. SD agar was buffered using 25 mM HEPES (pH 7.4). BPS, iron chelator; ferritin, 15 µg/ml ferritin. All plates were incubated for 4 days at 37°C under 5% CO2. Blue indicates pH values higher than 5.5. Green indicates pH values between 5.5 and 4. Yellow indicates pH values below 4. The assay was performed twice in duplicate. (6.36 MB TIF) [file ppat.1000217.s001.tif]

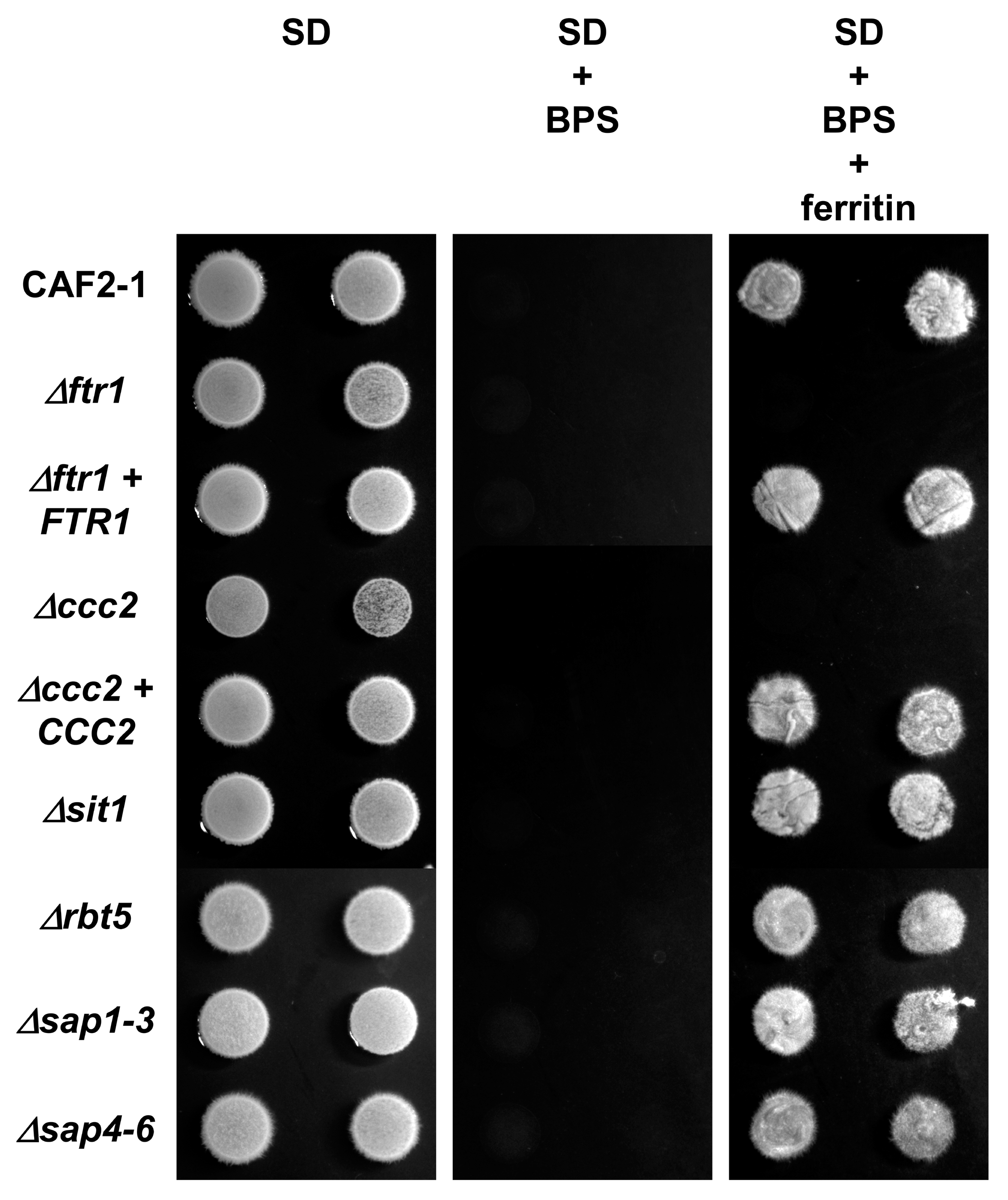

Supplement: Figure S2 — Examples of ferritin plates as described in Table 1. SD agar was buffered using 100 mM HEPES (pH 7.4). BPS, iron chelator. Ferritin, 5 µg/ml ferritin. All plates were incubated for 3 days at 37°C under 5% CO2. (2.68 MB TIF) [file ppat.1000217.s002.tif]

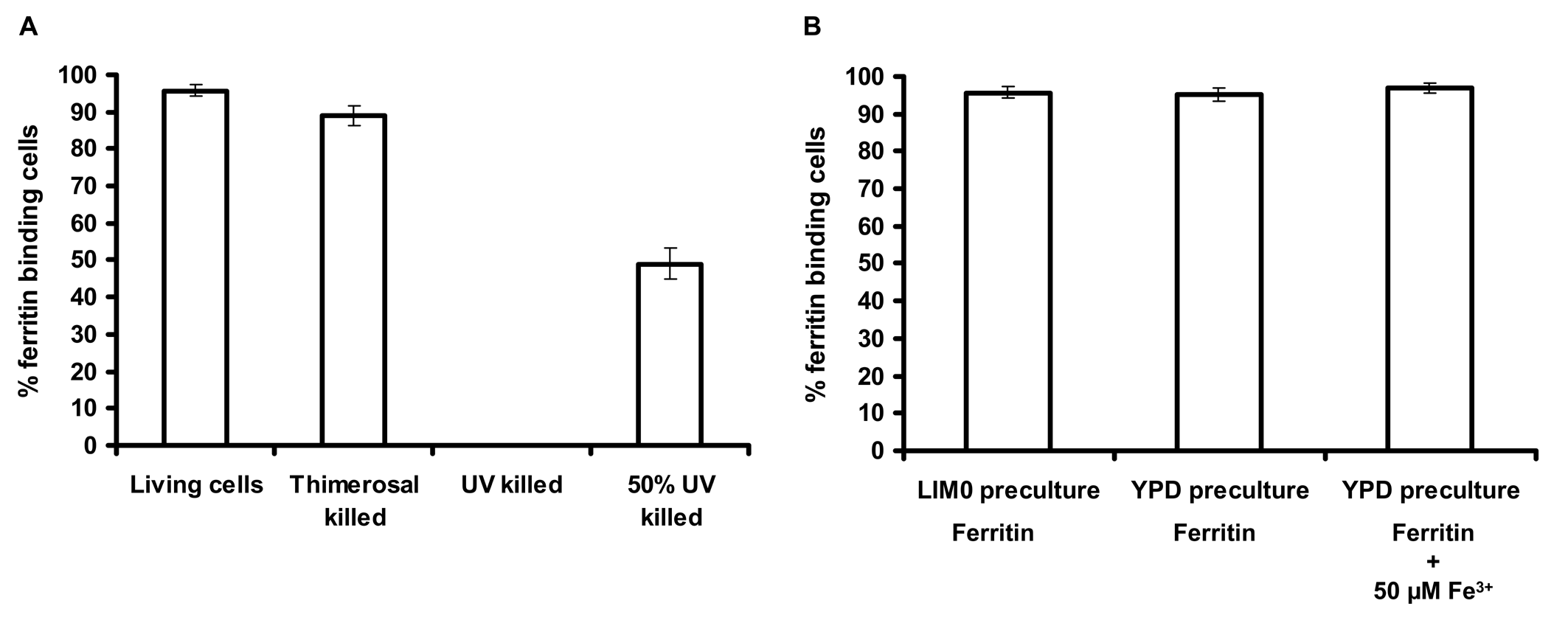

Supplement: Figure S3 — Ferritin binding does not require live cells or iron limitation and is UV sensitive. (A) Comparison of ferritin binding between live and dead cells (using thimerosal or UV light). (B) Cells from iron limitation medium (LIM0) or from YPD were used for the ferritin binding assay. Additionally, cells from the same YPD preculture were tested for ferritin binding with the addition of 50 µM iron chloride during the binding assay. (0.30 MB TIF) [file ppat.1000217.s003.tif]

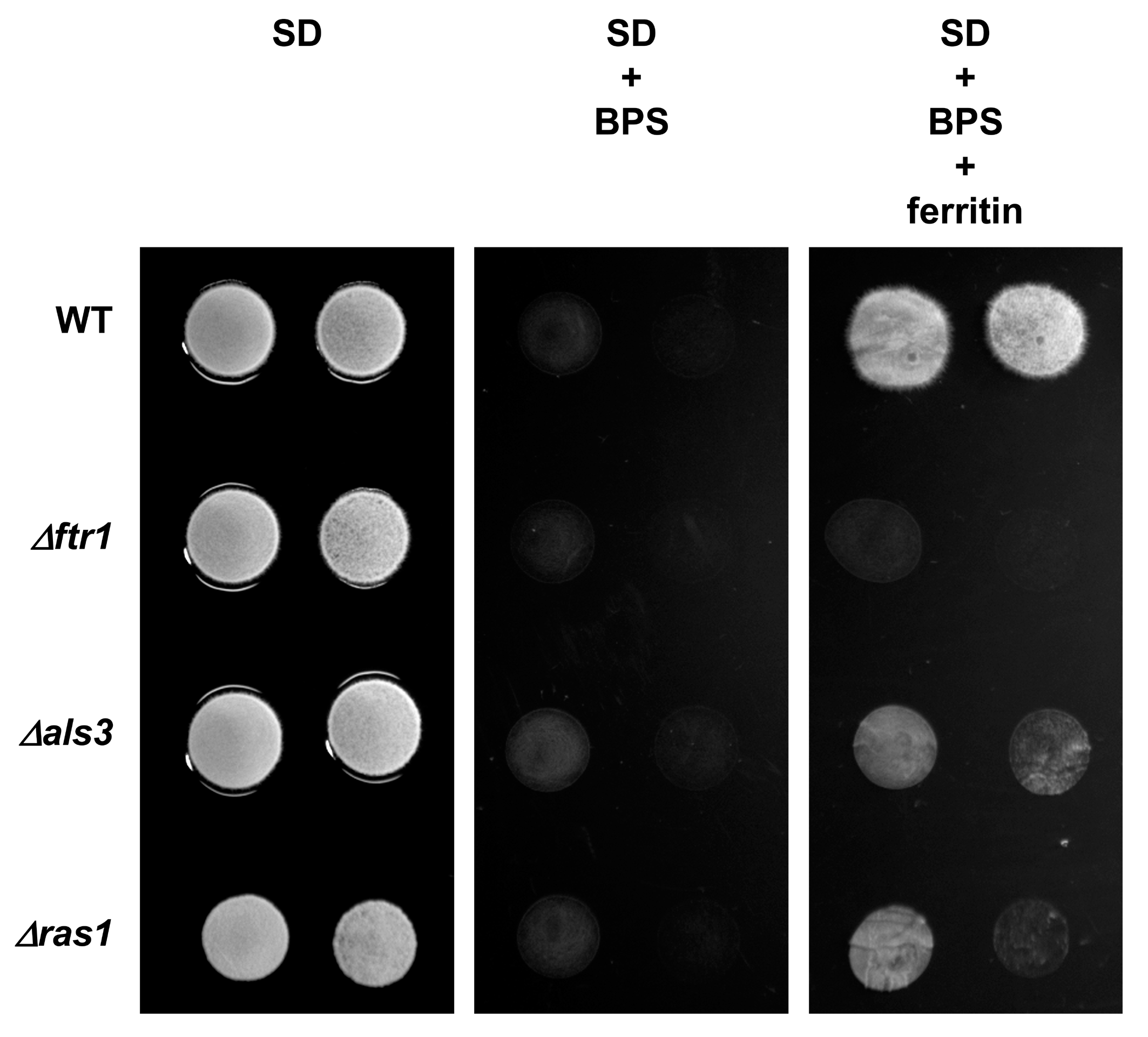

Supplement: Figure S4 — Growth of selected mutants on ferritin plates. SD agar was buffered using 100 mM HEPES (pH 7.4). BPS, iron chelator. Ferritin, 5 µg/ml ferritin. All plates were incubated for 3 days at 37°C under 5% CO2. (1.59 MB TIF) [file ppat.1000217.s004.tif]

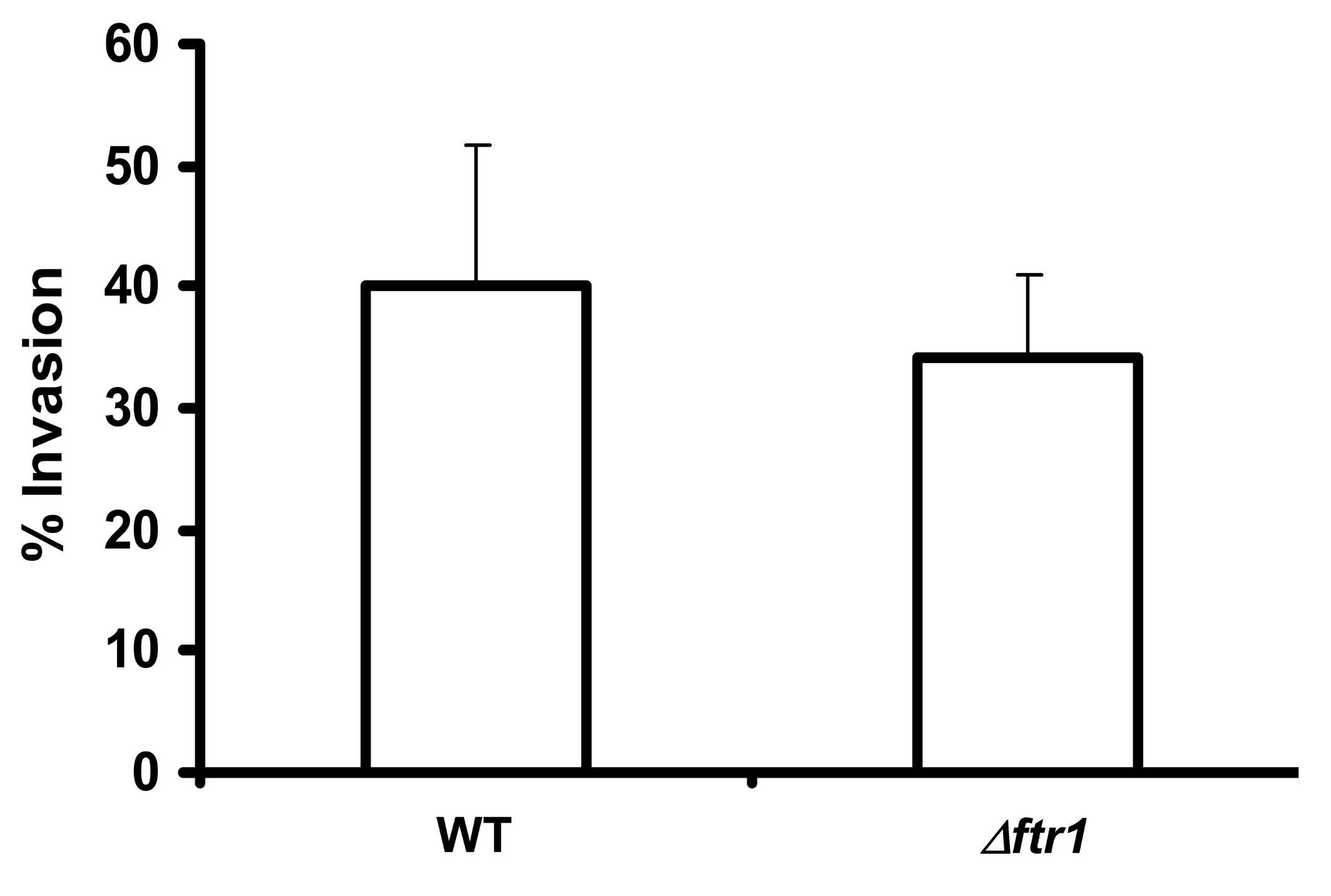

Supplement: Figure S5 — Invasion of epithelial cells by Δftr1. Aproximately 105 iron starved wild-type C. albicans cells (SC5314) or Δftr1 mutant cells were co-incubated with epithelial cells for 3 h. After fixation the samples were differentially stained and analysed under the fluorescence microscope. The experiment was performed three times in duplicate. No significant difference was observed between the wild-type strain and the Δftr1 mutant strain. (0.30 MB TIF) [file ppat.1000217.s005.tif]
